# Foxp2 Regulates Gene Networks Implicated in Neurite Outgrowth in the Developing Brain

**DOI:** 10.1371/journal.pgen.1002145

**Published:** 2011-07-07

**Authors:** Sonja C. Vernes, Peter L. Oliver, Elizabeth Spiteri, Helen E. Lockstone, Rathi Puliyadi, Jennifer M. Taylor, Joses Ho, Cedric Mombereau, Ariel Brewer, Ernesto Lowy, Jérôme Nicod, Matthias Groszer, Dilair Baban, Natasha Sahgal, Jean-Baptiste Cazier, Jiannis Ragoussis, Kay E. Davies, Daniel H. Geschwind, Simon E. Fisher

**Affiliations:** 1 Wellcome Trust Centre for Human Genetics, University of Oxford, Oxford, United Kingdom; 2 Medical Research Council Functional Genetics Unit, University of Oxford, Oxford, United Kingdom; 3 Program in Neurogenetics, Department of Neurology, University of California Los Angeles, Los Angeles, California, United States of America; 4 INSERM Institute du Fer à Moulin, University Pierre and Marie Curie, UMR-S 839, Paris, France; 5 Semel Institute and Department of Human Genetics, David Geffen School of Medicine, University of California Los Angeles, Los Angeles, California, United States of America; 6 Language and Genetics Department, Max Planck Institute for Psycholinguistics, Nijmegen, The Netherlands; University of Washington, United States of America

## Abstract

Forkhead-box protein P2 is a transcription factor that has been associated with intriguing aspects of cognitive function in humans, non-human mammals, and song-learning birds. Heterozygous mutations of the human *FOXP2* gene cause a monogenic speech and language disorder. Reduced functional dosage of the mouse version (*Foxp2*) causes deficient cortico-striatal synaptic plasticity and impairs motor-skill learning. Moreover, the songbird orthologue appears critically important for vocal learning. Across diverse vertebrate species, this well-conserved transcription factor is highly expressed in the developing and adult central nervous system. Very little is known about the mechanisms regulated by Foxp2 during brain development. We used an integrated functional genomics strategy to robustly define Foxp2-dependent pathways, both direct and indirect targets, in the embryonic brain. Specifically, we performed genome-wide *in vivo* ChIP–chip screens for Foxp2-binding and thereby identified a set of 264 high-confidence neural targets under strict, empirically derived significance thresholds. The findings, coupled to expression profiling and *in situ* hybridization of brain tissue from wild-type and mutant mouse embryos, strongly highlighted gene networks linked to neurite development. We followed up our genomics data with functional experiments, showing that Foxp2 impacts on neurite outgrowth in primary neurons and in neuronal cell models. Our data indicate that Foxp2 modulates neuronal network formation, by directly and indirectly regulating mRNAs involved in the development and plasticity of neuronal connections.

## Author Summary

Foxp2 codes for an intriguing regulatory protein that provides a window into unusual aspects of brain function in multiple species. For example, the gene is implicated in speech and language disorders in humans, song learning in songbirds, and learning of rapid movement sequences in mice. Foxp2 acts by tuning the expression levels of other genes (its downstream targets). In this study we used genome-wide techniques to comprehensively identify the major targets of Foxp2 in the embryonic brain, in order to understand its roles in fundamental biological pathways during neurodevelopment, which we followed up through functional analyses of neurons. Most notably, we found that Foxp2 directly and indirectly regulates networks of genes that alter the length and branching of neuronal projections, an important route for modulating the wiring of neural connections in the developing brain. Overall, our findings shed light on how Foxp2 directs particular features of nervous system development, helping us to build bridges between genes and complex aspects of brain function.

## Introduction

Forkhead-box protein P2 is a highly conserved vertebrate protein, belonging to an important group of transcription factors [1]. By modulating the expression of downstream target genes, forkhead-box proteins influence a diverse array of processes, including cell-cycle regulation, signal transduction, differentiation, patterning and metabolism [2]. They thereby play crucial roles during embryogenesis, in postnatal development and in the mature organism, and many have been linked to disease states [3]. The P subgroup is a divergent branch of forkhead-box proteins that share a distinctive DNA-binding domain located near the C-terminal end of the protein, as well as zinc-finger/leucine-zipper motifs that mediate dimerization, and a glutamine-rich region towards the N-terminus [4], [5].

Functional evidence from multiple species implicates Forkhead-box protein P2 in particularly intriguing aspects of brain development and function [1]. (Here we adopt the standard accepted nomenclature to refer to the protein in different species: FOXP2 in humans, Foxp2 in mice, FoxP2 in other chordates, with the corresponding gene names in italics [6].) In humans, damage to one copy of the *FOXP2* gene causes a rare neurodevelopmental disorder, characterised by difficulties mastering sequences of mouth movements during speech, as well as impaired language processing [4], [7], [8]. Heterozygous disruptions of the mouse orthologue (*Foxp2*) yield dramatic reductions in synaptic plasticity of cortico-striatal brain circuits, associated with deficits in learning of rapid motor skills [9]. Mouse pups with homozygous *Foxp2* mutations show more severe neural effects – gross motor impairments, delayed postnatal maturation of the cerebellum and dramatic reductions in emission of ultrasonic vocalisations – against a background of reduced weight-gain and postnatal lethality [9]-[11]. In addition, the avian orthologue (*FoxP2*) is required for normal vocal learning in songbirds [12], [13]. Selective knockdown of the gene in a key striatal nucleus in juvenile zebrafinches leads to incomplete and inaccurate imitation of tutor songs [14].

Studies of both human *FOXP2* and mouse *Foxp2* identified similarly strong CNS (central nervous system) expression during embryogenesis, which is confined to neurons (absent from glial cells) and enriched in various brain structures, including deep layers of the developing cortical plate, and parts of the striatum, thalamus and cerebellum [15],[16]. These embryonic expression patterns appear highly concordant in the different species, and show remarkable overlaps with sites of pathology identified by neuroimaging of human children and adults carrying *FOXP2* mutations [16], [17]. Neural expression of the gene continues postnatally and into adulthood [4], [15], and is also observed in certain non-neural tissues, most notably the distal alveolar lung epithelium, and the outflow tract and atrium of the cardiovascular system [18].

The above observations of well-conserved and specific CNS expression patterns [15], [16] suggest that Foxp2 is likely to have important functions in neurodevelopment. Nevertheless, as data continue to accumulate regarding its impacts on the postnatal brain [9], [11], [14], the specific roles of Foxp2 in the developing CNS remain largely elusive. One route for gaining insights into the biological processes controlled by a transcription factor is to define the regulatory networks that are directly downstream of it [1]. An efficient strategy for identifying direct targets exploits chromatin immunoprecipitation (ChIP) methods to screen the tissue of interest [19]. Two previous investigations have coupled ChIP with hybridisation to promoter microarrays (ChIP-chip) in order to uncover binding sites of FOXP2 in human foetal brain tissue [20] and in human neurons grown in culture [21]. Both screens were of limited scope – the microarrays in these studies comprised fragments from the 5′ ends of ∼5,000 loci [20], [21], representing a small percentage of the known gene promoters in the genome. Neither study combined ChIP data with large-scale expression analyses. A more recent report used mRNA expression profiling in human neuronal models transfected with different versions of FOXP2 to explore regulatory differences between the human and chimpanzee orthologues, but did not include any ChIP screening for direct targets [22].

In the present study, we performed a systematic large-scale *in vivo* ChIP-chip screen of the embryonic mouse brain, coupling Foxp2-ChIP to high-density arrays with oligonucleotides tiled across >17,000 promoters. We robustly established the empirical significance of our ChIP results in wild-type brains by determining the null distribution of signals generated by matched control tissue from littermates that expressed no Foxp2 protein. Under strict empirical thresholds that minimised false positive signals, we isolated a set of 264 high-confidence *in vivo* targets. Gene ontology (GO) analyses of the ChIP-chip data, as well as genome-wide expression profiling and *in situ* hybridisations of wild-type and mutant mice, converged on neurite outgrowth as one of the most prominent biological themes associated with Foxp2 function in the embryonic CNS. We went on to directly demonstrate, using neuronal cell models and primary neurons from the embryonic mouse brain, that Foxp2 alters expression of neurite-outgrowth targets and thereby influences neurite process length and branch number.

## Results

### Genome-wide identification of *in vivo* Foxp2 targets in embryonic mouse brain

**Figure 1:**
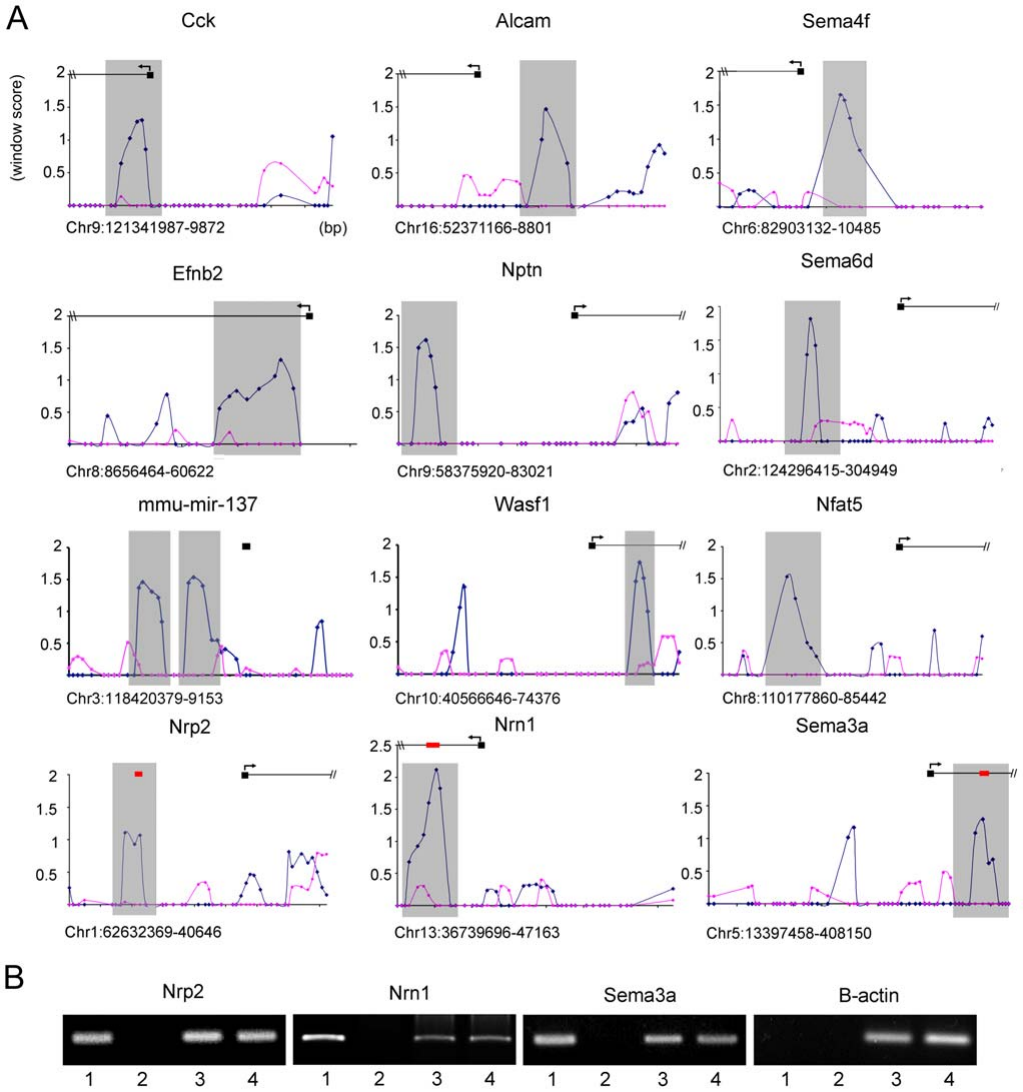
*In vivo* Foxp2 promoter occupancy in embryonic mouse brain. (A) Foxp2-ChIP window enrichment scores of probes across promoters of a subset of putative targets from the neurite outgrowth and axon guidance pathways. The window score (Y axis) is given versus the distance across the promoter region (X axis) - each cross bar represents 1000 bp and each data point represents a single probe (chromosome and position in bp are given below the X axis of each graph). The enrichment in the wild-type experiments is shown by the blue trace, and the pink trace indicates the corresponding values in null mutant controls that lack Foxp2 protein. The predicted start site of the gene (as annotated on UCSC genome browser: http://genome.ucsc.edu/) is given by the black box and arrows denote the direction of transcription. Grey shading indicates the peak area of enrichment and the most likely region for Foxp2 binding to occur. (B) Analysis of *in vivo* promoter occupancy. DNA isolated via Foxp2-ChIP was PCR amplified using primers directed towards the promoter regions of *Nrp2*, *Sema3a*, *Nrn1* or the *β-actin* control. The position of these amplicons within the target promoter region is given by red bars in part A. Results from E16 wild type mice were compared to those from homozygous null mutant littermates. Lane 1 =  wild-type Foxp2-ChIP, lane 2 =  mutant null Foxp2-ChIP, lane 3 =  wild-type total DNA, lane 4 =  mutant total DNA. Target gene promoters were found to be specifically enriched in Foxp2-ChIP samples isolated from wild-type brains compared to those from null mutant brains, unlike the *β-actin* control promoter. Gels are representative of results from triplicate experiments.

*In vivo* Foxp2-ChIP screening was carried out using brains harvested from embryonic mice. Experiments were performed with mice that were wild-type for Foxp2, as well as homozygous littermates that do not express any Foxp2 protein (*Foxp2-S321X* mutants; see Materials and Methods) [9]. The different types of sample were screened in parallel, undergoing identical experimental manipulations and data processing. In this context, the homozygous mutant mouse tissue acts as an ideal negative control [21]. Since such samples completely lack Foxp2 protein (see Figure S1 and [9]), fragments that are pulled down by Foxp2-ChIP in these cases give an unbiased empirical indication of background noise and false positive rates yielded by the procedure. Whole mouse brains from wild-type or mutant mice were harvested at embryonic day 16 (E16), corresponding to a timepoint at which particularly high levels of Foxp2 expression are observed in the developing CNS [16]. Chromatin isolated by Foxp2-ChIP was labelled and hybridised to DNA microarrays covering the promoter regions of ∼17,000 mouse transcripts (Agilent Technologies), using total input DNA as a reference sample. Each promoter on these arrays is represented by an average of twenty-five 60-mer probes spanning ∼5.5 kb upstream and ∼2.5 kb downstream of the transcription start site, allowing peak regions of binding to be precisely defined (Figure 1). Moreover, the presence of multiple probes for each promoter scattered throughout the array gives independent enrichment values within the same promoter, which aids discrimination of real biological targets from false positive events. Specifically, since the shearing process during ChIP produces overlapping fragments of chromatin, true targets should show evidence of enrichment for multiple probes across the promoter region, while promoters with only a single enriched probe are most likely to be false positive results.

In order to identify enriched promoters, Foxp2-ChIP data were analysed as per Materials and Methods. Briefly, array data from independent biological replicates (three independent ChIP experiments hybridised to one each of three array sets) were normalised for each genotype (wild-type or mutant control) separately. Normalised array data (excluding probes with a negative average enrichment across replicate experiments) were subjected to a sliding window analysis, using a similar method to that employed in genome-wide ChIP-chip studies of other forkhead transcription factors [23]. Each probe was assigned a value (window-adjusted score) based on the median fold enrichment of itself and its neighbouring probe on either side (within 500 bp upstream and 500 bp downstream), and then probes were ranked based on this window score.

By analysing the distribution of window scores observed in the mutant null control experiments we were able to derive an empirical threshold for significance, which could then be applied to the wild-type data. We found that window scores greater than or equal to 0.974 (corresponding to ∼2-fold enrichment) excluded 99% of the data-points in the mutant null control experiments. When we applied this threshold to data from wild-type experiments, we identified a set of 1,217 promoter regions that were consistently enriched by Foxp2-ChIP over 3 replicates in wild-type mouse brains (Table S1). On inspection of the locations of the enriched probes throughout the mouse genome, no positional bias was observed (Figure S2). Since some of the enriched regions lay close to the transcriptional start site (TSS) of more than one gene, the 1,217 promoter regions corresponded to 1,253 genes. Of note, using the same analysis parameters, only 147 genes were enriched in the mutant null controls, suggesting a low false discovery rate. Nevertheless, in order to minimize false-positive findings, we excluded any enriched genes from the wild-type dataset that also had window scores exceeding the 99% threshold in the mutant control dataset. This filtering process yielded a slightly smaller set of 1,164 putative targets (Table S2).

When we applied stricter thresholds to the wild-type data, selecting only those promoters in which at least one probe gave a window score of ≥ 1.5, we identified a shortlist of 259 promoter regions. Since a small number of peak regions lay directly between the TSSs of two different genes, these 259 promoters corresponded to a slightly higher total of 266 genes. Crucially, the same analyses of the entire mutant null control dataset identified only a single gene in the genome with a window score of ≥ 1.5 (the *Pigt* gene), indicating an extremely low rate of false positives under these stricter selection criteria. We excluded two genes from the strict wild-type shortlist (*Pigt* and *Zfp496*) since they contained probes that exceeded the 99% threshold (i.e. window score of >0.974) in mutant null controls (Figure S3). The outcome of these analyses was a final curated shortlist of 264 high-confidence *in vivo* targets (Table S3).

Given that DNA is sheared randomly during the ChIP process, we would expect a true Foxp2 binding event to be represented by a peak of enrichment at a target promoter. This peak would result from the sheared DNA forming a series of overlapping fragments, with the region closest to the binding site showing the highest degree of enrichment (i.e. highest number of fragments pulled down during immunoprecipitation) and with progressively less enrichment observed as the distance to the binding site increases on either side. Figure 1A gives typical examples of the enrichment peaks observed for putative targets from our Foxp2-ChIP dataset. Examination of corresponding data from mutant control experiments emphasises the relative lack of enrichment in nulls that lack Foxp2 protein, indicating that the enrichment in wild-type samples results from highly specific Foxp2-mediated interactions. Furthermore, we followed up a subset of candidates with qPCR, consistently confirming their enrichment (Figure 1B).

Enriched regions represented in the shortlist of high-confidence targets were assessed *in silico* for any over-represented sequence motifs (see Text S1). This analysis did not enforce *a priori* conditions of motif sequence, other than a length restriction of 8 bases. This meant that rather than limiting our search to occurrences of known patterns in the promoters, we obtained an unbiased list of motifs that were characteristic of the Foxp2-ChIP target promoters. Eight sequences (motifs A-H) were found to be significantly over-represented (p<0.05) in the shortlist of high confidence target promoter sequences (Table 1). Importantly, the three most commonly identified over-represented motifs from this unbiased search (A–C) were partial or complete matches to well established FOX/FOXP/FOXP2 binding motifs (RYMAAYA/TATTTRT/AATTTGT), providing additional strong support for the biological relevance of our findings. A further over-represented motif (motif D) did not match the known consensus motifs and was detected in 182 promoters out of the 247 promoter regions that could be surveyed from the Foxp2-ChIP shortlist (See Text S1; Figure S4A). Thus, we reasoned that it may represent a novel putative Foxp2 binding sequence. EMSA (Electrophoretic Mobility Shift Assay) experiments demonstrated strong specific binding of FOXP2 to this motif (Figure S4B), when located in putative Foxp2 target promoter sequences, such as those for *Nrn1, Nfat5* and *Sema6d*. However, not all occurrences of this motif were strongly bound by Foxp2, suggesting that while the site is capable of being bound by Foxp2 protein, the binding is context specific – as is regularly seen for other FOX family binding sites [24].

**Table 1:** Over-represented motifs identified in the shortlist of Foxp2-ChIP target promoters.

Motif	Sequence	p-value	No. of promoters
A	A[A/G][G/T]TA[A/C][C/T]T	2.79e-08 to 3.59e-02	206
B	A[A/T]GTAAAT	7.42e-05 to 2.33e-02	195
C	TTACTTA[A/T]	1.7e-03 to 1.39e-02	187
D	AAAG[G/C]AAA	9.6e-03 to 4.36e-02	182
E	AGTAA[T/A]TG	5e-04 to 2.44e-02	138
F	A[T/A]T[C/G]A[T/A]TT	1.46e-02 to 3.29e-02	150
G	ATTACTAA	1.62e-02	124
H	AATACATT	4.27e-02	138

In addition to the use of *in vivo* ChIP to uncover target genes that are directly bound by Foxp2 (direct targets), we assessed regulatory cascades further downstream (indirect targets) via an expression profiling approach. Again we focused on E16 mouse brain tissue, analysing the same genotypes (wild-type mice and their homozygous *Foxp2-S321X* littermates, 5 and 6 biological replicates, respectively) on the same genomic background as used for the ChIP experiments. While ChIP identifies DNA-binding events of Foxp2-positive cells, expression profiling is expected to be more sensitive to tissue heterogeneity. Therefore we selected a key site of high Foxp2 expression with considerable prior evidence of functional relevance [9], [14]–[17], the ganglionic eminences (developing striatum and pallidum). Analysis of genome-wide expression data (see Materials and Methods for details) identified 340 genes that were differentially expressed (p<0.01) between wild-type and *Foxp2-S321X* homozygous mutant samples (Table S4). 180 of these genes showed reduced expression in absence of Foxp2 protein, while the remaining 160 genes showed increases (Table S4). Of these 340 genes, 19 genes (5.6%) were found in common with the ChIP-chip target gene list (Table S5), including those with known CNS functions, such as *Nell2* (neural epidermal growth factor-like like 2), *Nrn1* (neuritin), *Cck* (cholecystokinin), and *Alcam* (activated leukocyte cell adhesion molecule). Notably, the human orthologues of *Nrn1* and *Cck* have been independently proposed as top direct targets in small-scale ChIP screens of human foetal tissue [20].

**Figure 2:**
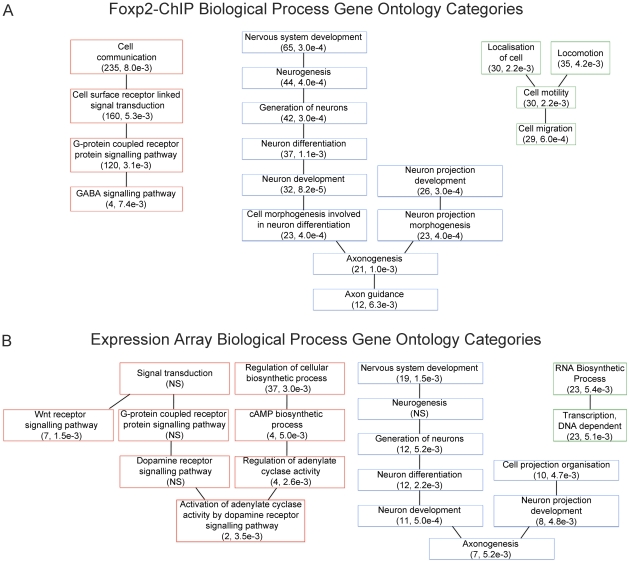
Gene Ontology categories suggest Foxp2 involvement in known functional pathways. (A) Gene ontology analysis of Foxp2-ChIP target gene list (see Table S2) and (B) Gene ontology analysis of genes differentially expressed between *Foxp2-S321X* and wild-type embryos (see Table S4). For each, a selection of biological process ontology categories are shown that were found to be significantly over-represented in the putative target gene lists, including neurogenesis, neuron projection development and axonogenesis (A–B), cell migration (A) and G-protein receptor signalling (B). The number of genes per category and p-values are shown in brackets.

We went on to determine whether any biological themes were over-represented within the direct targets (promoter bound by Foxp2) and indirect targets (not bound by, but regulated downstream of Foxp2), using unbiased GO analyses. The Foxp2-ChIP and expression profiling datasets were each assessed independently using the WebGestalt program [25], identifying functional categories that were significantly enriched (Figure 2 and Figure S5). In the Foxp2-ChIP dataset we observed significant over-representation of genes involved in processes including cell motility and migration, chromatin architecture and assembly, synaptic transmission, and a number of categories associated with RNA metabolism such as regulation of RNA stability and mRNA processing. In the expression profiling dataset significant categories included regulation of transcription, actin cytoskeleton organisation and biogenesis, and cellular protein catabolism. Consistent with previous studies [20], [21], nervous system development, neurogenesis and multiple G-protein signalling categories — including G-protein coupled receptor signalling (ChIP), and G-protein signalling and Wnt receptor signalling (expression) — were significant in both datasets.

**Figure 3:**
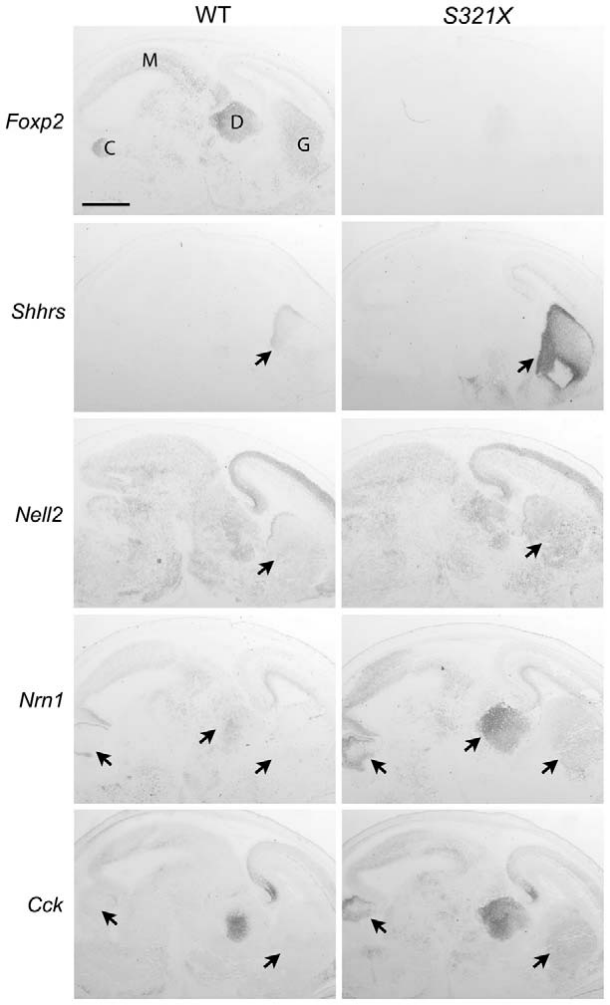
Foxp2 regulates putative target gene expression in the E16 mouse brain. *In situ* hybridisation of wild-type (WT) and *Foxp2-S321X* homozygous mutant brains at E16. *Foxp2* is predominantly expressed in the ganglionic eminences (G), diencephalon (D), midbrain (M) and developing cerebellum (C) in WT mice. Figure displays results from selected transcripts that had shown increased levels in expression profiling of Foxp2 mutant mice. *Nrn1*, *Nell2* and *Cck* were also identified from the ChIP screen. Arrows indicate equivalent regions from brains of both genotypes. In *Foxp2* mutants, all target genes show increased expression in the ganglionic eminence, with additional increased expression of *Nrn1* in the diencephalon and *Nrn1* and *Cck* in the developing cerebellum. Scale bar is 1 mm. Results are representative of 3 mice of each genotype.

We next performed *in situ* hybridisation on brains from wild-type and *Foxp2-S321X* E16 embryos, to further assess major targets suggested by the ChIP and expression profiling screens. Consistent with previously published data [16], in addition to the developing striatum, Foxp2 expression at this developmental stage is highest in the diencephalon (developing thalamus), midbrain and cerebellar primordium (Figure 3). The *in situ* hybridisation data confirmed regulation of *Shhrs* (also known as *Dlx6as1* or *Evf1/2*), a transcript showing greater than 200-fold increased expression levels in *S321X* mice. This noncoding RNA is highly specific to the ganglionic eminences in the embryo and is known to play a vital role in the control of the homeodomain transcription factors Dlx5 and Dlx6 [26], [27]. These data illustrate that loss of *Foxp2* can influence transcripts central to key neurodevelopmental processes *in vivo*. We then focused on target genes common to both ChIP and expression profiling datasets, to determine whether expression changes could be observed, not only in the ganglionic eminences, but also elsewhere in the developing brain (Figure 3). Indeed, *Nell2*, *Nrn1* and *Cck* all demonstrated clear increases in expression in the developing basal ganglia at E16 in the Foxp2 mutant compared to wild-type, in agreement with the array data (Figure 3 and Figure S5), providing further evidence that they are indeed direct targets, repressed by Foxp2. Significantly, *Nrn1*, a gene important for neuronal outgrowth [28], showed strongly increased expression in mutants in additional regions where *Foxp2* is typically expressed, including the developing thalamus and cerebellum (Figure 3). Similarly, *Cck* shows additional increases in expression in the cerebellum (Figure 3).

Certain putative direct Foxp2 targets with known roles in the CNS, such as *Ywhah* (tyrosine 3-monooxygenase/tryptophan 5-monooxygenase activation protein, eta polypeptide) and *Wasf1* (WASP family 1), are ubiquitously expressed in the developing mouse brain [29]. However, other genes are thought to have more localised and/or temporally defined patterns of expression. To ascertain whether these targets are co-expressed with *Foxp2*, further *in situ* hybridisation was carried out at E16. The results demonstrate that genes from the ChIP dataset with established CNS functions are found in a range of *Foxp2*-expressing brain regions (Figure S6).

### Foxp2 regulates neurite outgrowth in neurons

**Figure 4:**
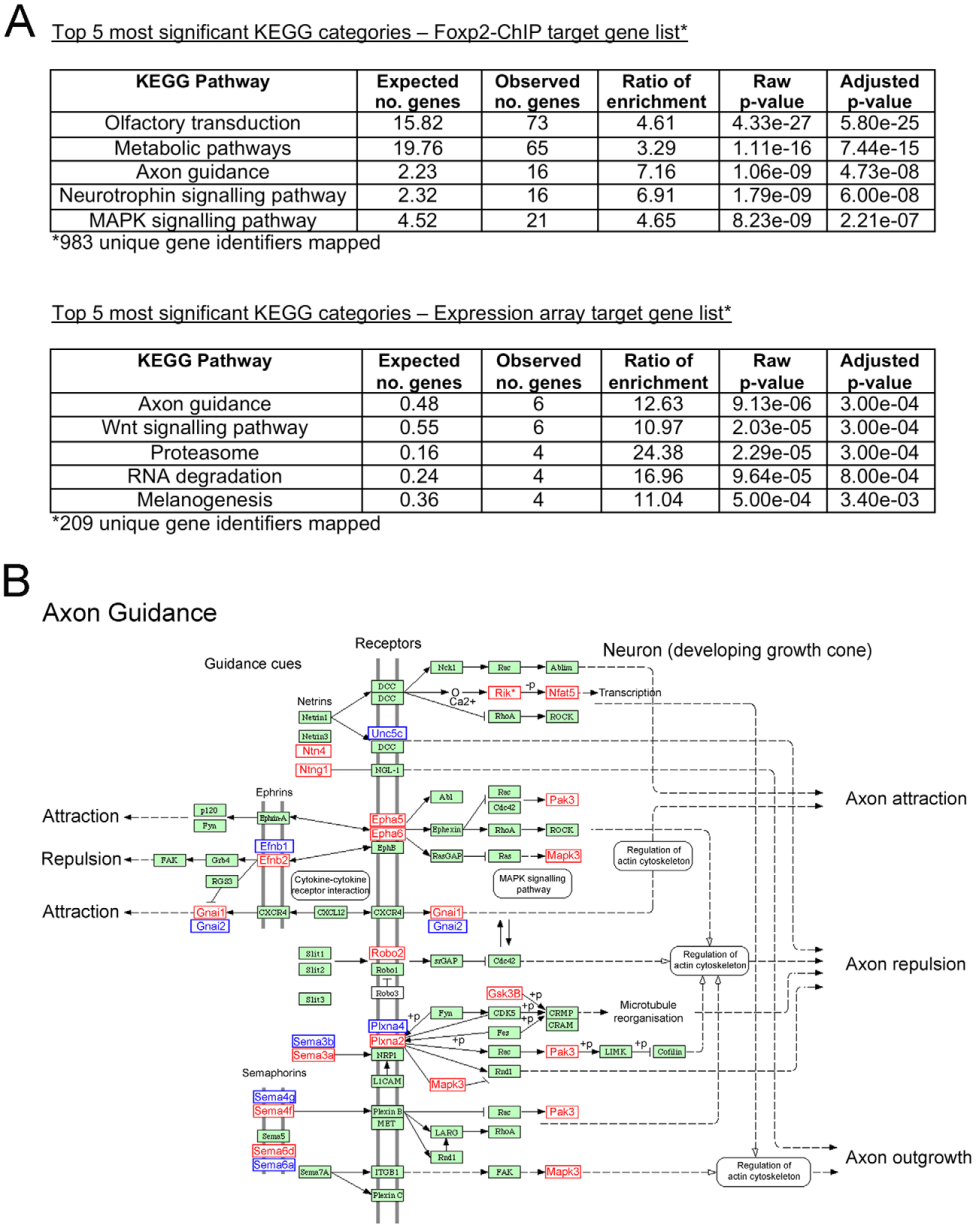
Neurite outgrowth and axon guidance pathways are major biological themes of Foxp2-dependent networks in embryonic brain. (A) The Foxp2 target gene datasets obtained via ChIP-chip (see Table S2) and expression profiling (see Table S4) were assessed separately for significantly over-represented KEGG pathways. (B) The axon guidance pathway displayed one of the most highly significant over-representations in the respective datasets (Foxp2-ChIP: p = 4.73×10^−8^ and expression profiling: p = 3.00×10^−4^). Genes that were present in this pathway from the Foxp2-ChIP target list (window score >0.974) are highlighted on the WebGestalt KEGG pathway in red, with those from expression analysis (p<0.01) highlighted in blue. None of the genes shown were common to both datasets. Expression profiling showed *Efnb1* and *Plxna4* to be upregulated *in vivo* in response to Foxp2; all other genes highlighted in blue were downregulated. Figure adapted from the WebGestalt program results (see Materials and Methods).

The GO analyses of independent Foxp2 target data from genome-wide ChIP and expression profiling uncovered a consistent and statistically significant over-representation of genes involved in neurite development & morphogenesis, axon extension and axon guidance pathways (Table 2). Multiple GO categories associated with such processes were significantly over-represented in at least one of the datasets, and several of these functional classes were significant in both datasets including neurogenesis, neuron projection development and axonogenesis (Figure 2 and Table 2). Furthermore, when we investigated KEGG pathways associated with these datasets, we observed enrichment of genes in a number of pathways (Figure 4A and Table S6), one of the most significant of which was the axon guidance pathway (p = 4.73×10^−8^ and p = 3.00×10^−4^ in Foxp2-ChIP and expression profiling datasets, respectively). Interestingly, a number of different but interacting genes within this pathway were identified in the two datasets (Figure 4B), suggesting that direct and indirect targets may represent different aspects of the same functional downstream effects of Foxp2-mediated regulation. In sum, our unbiased genomic screens for Foxp2-dependent gene networks predicted that neurite outgrowth and axon guidance should be key biological themes associated with Foxp2 function in the developing brain.

**Table 2:** GO categories significantly over-represented in Foxp2-ChIP (Table S2) and expression profiling (Table S4) datasets.

Gene Ontology Category	Foxp2-ChIP	Expression arrays
Nervous system development	3.00e-04	1.50e-03
Generation of neurons	3.00e-04	5.20e-03
Neurogenesis	4.00e-04	-
Regulation of neurogenesis	4.20e-04	-
Negative regulation of neurogenesis	2.00e-03	-
Neuron development	8.21e-05	5.00e-04
Neuron differentiation	1.10e-03	2.20e-03
Cellular morphogenesis involved in differentiation	-	1.20e-03
Cell projection	3.66e-02	9.10e-03
Cell projection organization	-	4.70e-03
Negative regulation of cell projection organization	4.00e-04	-
Cell projection morphogenesis	1.80e-03	-
Neuron projection development	3.00e-04	4.80e-03
Neuron projection morphogenesis	4.00e-04	-
Regulation of neuron projection development	2.00e-03	-
Axonogenesis	1.00e-03	5.20e-03
Axon guidance	6.30e-03	-

**Figure 5:**
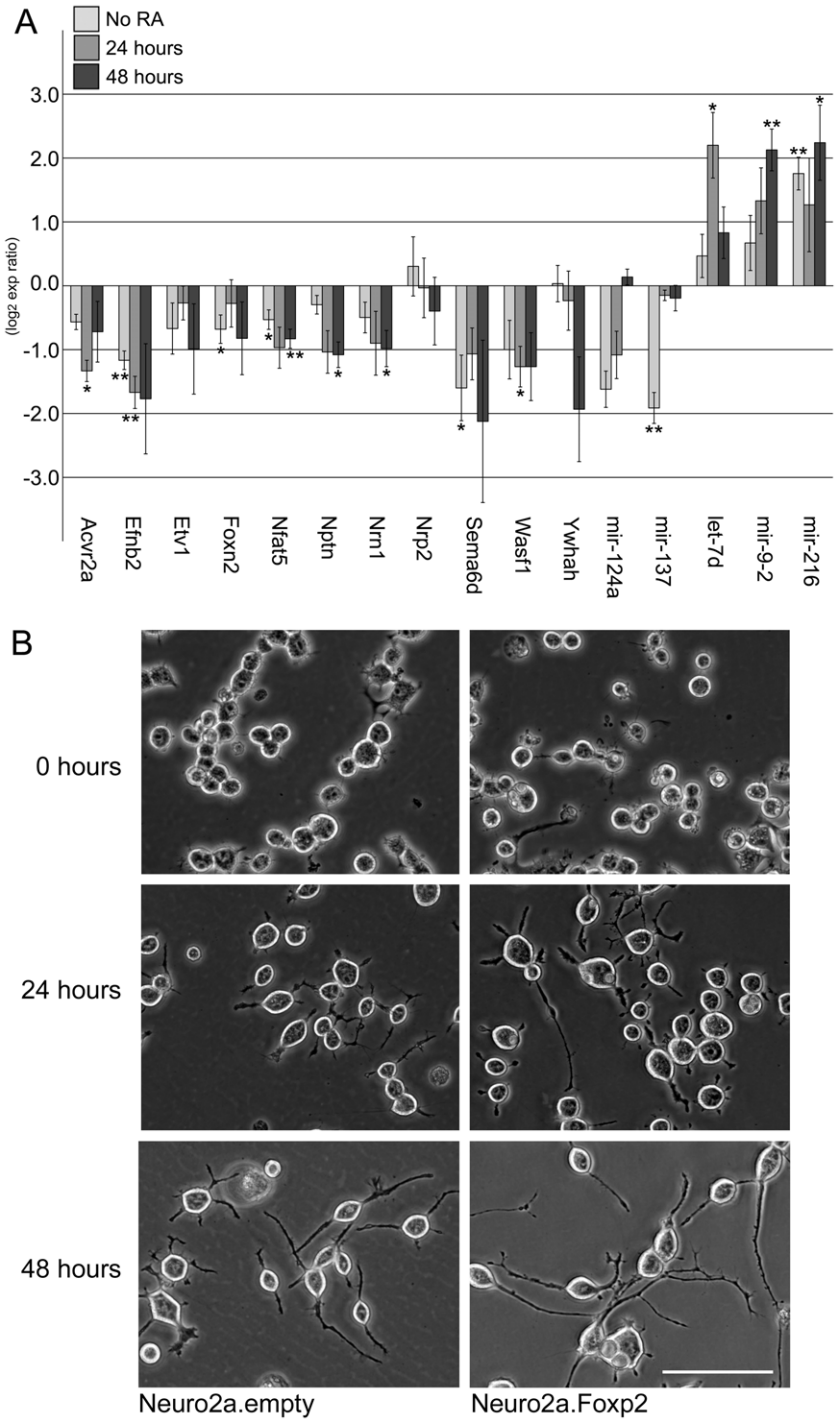
Foxp2 modulates neurite outgrowth in neuronal cell models. (A) Expression of target genes involved in neurite outgrowth and associated pathways. Quantitative PCR was performed for cDNA reverse-transcribed from stable Neuro2a cell-lines undergoing no differentiation or following 24 or 48 hours differentiation (via the addition of retinoic acid, RA). Expression changes are mean log_2_ expression ratios comparing three independent clones stably transfected with Foxp2 and three independent clones stably transfected with an empty vector, normalized for equal expression of the internal control, *Gapdh,* or *U6* (for miRNAs). Error bars indicate the ± SEM, p-values were calculated using a two-tailed unpaired t-test (** = p<0.01, * = p<0.05). (B) Cells expressing Foxp2 demonstrate visible changes in neurite outgrowth. Neuro2a cells stably transfected with Foxp2 or an empty vector control were grown for 24 and 48 hours in differentiation media before images were captured directly under a light microscope. Pictures are representative of >15 images taken for three independent clones of each cell type (Scale bar, 100 µm).

We went on to test this prediction through genetic and functional analyses of neuronal cell models and primary neurons. First, we independently assessed whether differences in Foxp2 expression affect expression of putative direct target genes involved in neurite outgrowth, using a well-validated murine cell model. Neuro2a cells are neuron-derived cells that can be differentiated to take on a more neuron-like identity via exposure to retinoic acid. These cells were stably transfected with *Foxp2* or with an empty vector control, and then grown in media either with or without the addition of retinoic acid. Cells that were over-expressing Foxp2 demonstrated consistent expression changes for multiple direct target genes that were identified in our E16 ChIP screen and implicated in neurite outgrowth pathways (Figure 5A). Significant repression of target gene expression was observed both pre- and post-differentiation; however most of the neurite-outgrowth genes showed greater fold changes following differentiation. Next, we formally assessed the hypothesis that changes in Foxp2 levels, and the concomitant alterations in expression of neurite-outgrowth genes, yield detectable differences in the growth of neurites in these cells. After 24 and 48 hours of retinoic acid treatment (matching the timepoints for analyses target gene expression) we performed blind scoring of cells and observed striking qualitative differences in neurite outgrowth when cells over-expressed Foxp2, as compared to sham-transfected controls. Cells that had been transfected with Foxp2 prior to differentiation consistently displayed increased neurite length, in a manner that was easy to distinguish from controls (Figure 5B).

To further assess the *in vivo* relevance of these findings, we examined whether there were corresponding phenotypic effects mediated by functional Foxp2 in neurons of the developing brain. We isolated primary neurons from the ganglionic eminences of E16 mouse brains, matching the region and timepoint used for our original target screening. Here, we aimed to directly test whether the Foxp2-positive neurons derived from the developing basal ganglia show altered neurite outgrowth when the gene is mutated. The assay was facilitated by availability of a mouse model (*Foxp2-R552H*) in which the protein is expressed at normal levels, but is nevertheless dysfunctional [9]. *R552H* mice recapitulate an aetiological mutation that causes speech and language deficits in a large human family. This change yields a substitution in the DNA-binding domain which severely impairs the transcription factor function of the mutant protein [30], such that the overall phenotype of homozygous *R552H* animals is very similar to that observed for mice which completely lack Foxp2 [9]–[11]. However, unlike the Foxp2-null mice, *R552H* homozygotes still express detectable levels of the protein, allowing us to clearly identify Foxp2-positive cells in our primary cultures via antibody staining. This represents an important measure, given the heterogeneous nature of the dissected material used to generate the primary culture.

**Figure 6:**
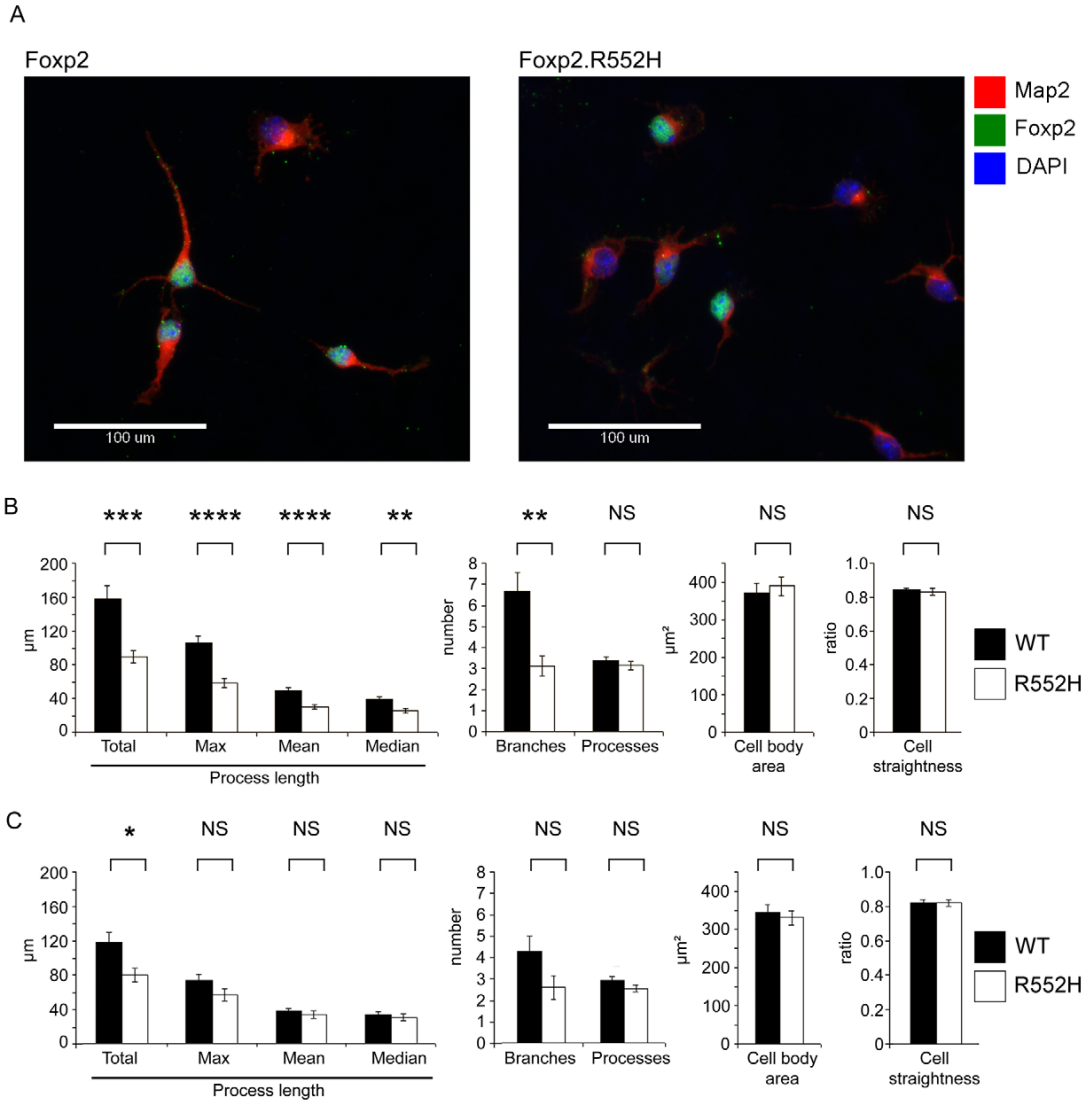
Foxp2 regulates neurite outgrowth in primary neurons. (A) Primary cells were harvested from the ganglionic eminences (developing striatum and pallidum) of wild-type and homozygous *Foxp2-R552H* E16 littermates and grown in culture for 4 days before fixation and staining for immunofluorescence. Map2 was detected using a TRITC-conjugated (red) antibody and Foxp2 via a FITC-conjugated (green) antibody. The R552H mutation produces a form of Foxp2 protein that appears to be largely non-functional *in vitro* and *in vivo (*see references [30] and [9], respectively), but is still detectable via immunofluorescence. DAPI counterstain (blue) indicates the location of nuclei. (Scale bar, 100 µm) (B–C) Quantification of properties of neurite outgrowths in primary culture. Neurites were quantified for all cells in each image and then the data were separated into Foxp2-positive and Foxp2-negative cells. These data represent the mean of 141 Foxp2-positive cells taken from wild-type embryos and 84 Foxp2-positive cells taken from R552H littermates (B) and the mean values of Foxp2-negative cells taken from the same wild-type embryos (142 cells) and the same homozygous R552H littermates (115 cells) (C). Cells were harvested from six wild-type and four *Foxp2-R552H* embryos, across two litters. All measures of process length as well as the number of branches per cell showed significant differences between wild-type (black bars) and *Foxp2-R552H* mutant (white bars) mice. However no significant contribution was observed for embryo for any measure except ‘Processes’ (p = 0.018), a measure that was not significant between genotypes. Error bars indicate the ± SEM and p-values were calculated using ANCOVA followed by post-hoc Sidak correction (**** = p<0.001, *** = p = 0.001, ** = p<0.01, * = p<0.05).

We again observed obvious differences in neurite outgrowth associated with presence of functional Foxp2 (Figure 6A). A blinded analysis revealed statistically significant changes in quantitative measures of neurite outgrowth for Foxp2-expressing neurons from wild-type embryos as compared to those from homozygous *Foxp2-R552H* littermates (Figure 6B). In particular, the latter showed significant reductions in total outgrowth (p = 0.001); mean (p<0.001), median (p = 0.008) and maximum process length (p<0.001); and average number of branches (p = 0.003). Thus, loss of Foxp2 function in striatal neurons that normally express this transcription factor yields significant reductions in multiple indices of neurite outgrowth. When Foxp2-negative cells from the wild-type cultures were compared to equivalent cells from mutants (Figure 6C), it was only the total outgrowth that met significance (p = 0.013).

These findings are strongly in agreement with differences in levels of Foxp2 expression, neurite outgrowth and correlated physiological properties between the two major subpopulations of striatal medium spiny neurons (MSNs) *in vivo*. While both striatonigral (Drd1a) and striatopallidal (Drd2) MSNs continue to increase their dendritic area well into adulthood, Drd1a MSNs develop significantly more dendrites [31]. This dichotomy in dendritic growth contributes to key physiological differences between both MSN populations, although the underlying mechanisms remain unknown [31]. Furthermore, studies of cultured striatal neurons demonstrate that Drd1a MSNs have larger dendritic trees than Drd2 MSNs, invoking intrinsic mechanisms [31]. To study whether these intrinsic differences in dendritic growth correlate with Foxp2 expression levels, we investigated mice expressing enhanced green fluorescent protein (EGFP) either mainly in Drd1a or Drd2 MSNs [32]. We found that Foxp2 shows consistently high expression in striatonigral Drd1a MSNs and very low expression in Drd2 MSNs throughout the striatum (Figure S7), further supporting roles for Foxp2 in neurite outgrowth.

## Discussion

Although early studies of Foxp2 orthologues in multiple species suggested that it may play crucial roles in neurodevelopment [15], [16], the exact nature of such roles has not been established. Indeed, much of the existing knowledge regarding neuronal functions of this transcription factor instead concerns its impacts on the postnatal CNS [9], [14]. In the present study we employed a high-throughput functional genomic strategy to shed new light on the *in vivo* activities of *Foxp2*-dependent pathways in the developing CNS.

Of note, among the biological themes that we identified, our comprehensive ChIP-chip and expression profiling in midgestation brain tissue independently and consistently highlighted gene networks underlying neurite development and morphogenesis, axon extension and axon guidance. These findings drove us to specifically assess the impact of the *Foxp2* gene on neurite outgrowth phenotypes in genetically manipulated neuronal cell models and primary neurons from embryos of mutant mice. Our functional experiments confirmed regulation of the highlighted gene networks and indicated that wild-type *Foxp2* thus enhances multiple facets of neurite development *in vivo*, including outgrowth process length and branch number. The data suggest that the mode of action may be predominantly cell autonomous, since the functional effects were mainly restricted to the subset of Foxp2-expressing cells within a mixed population of neurons. This possibility of cell-autonomous effects is an interesting hypothesis that could be clarified in further studies.

Our neurite outgrowth findings are in line with new evidence regarding the functional impact of evolutionary differences between FOXP2 orthologues [33]. For example, it is known that this transcription factor underwent two amino-acid substitutions on the human lineage after splitting from the chimpanzee lineage, leading to speculation that such changes may have been important for evolution of spoken language. In a recent study, researchers inserted the relevant substitutions into the endogenous *Foxp2* gene of mice, and observed that striatal neurons had significantly longer dendrites and increased synaptic plasticity [33]. By contrast, we have shown that mice with loss of function of Foxp2 have statistically significant reductions in neurite outgrowth (Figure 6 in the present paper) and decreased synaptic plasticity [9].

Furthermore, the identification of potential regulatory links between *Foxp2* and neural connectivity may be informative for wider discussions regarding the evolution of vocal learning [34]. Auditory-guided vocal learning is a rare trait that is only found in a small number of animal groups; the best understood examples include speech acquisition in humans and learning of song by certain bird species. As noted above, while human *FOXP2* has been implicated in speech abilities [4], [7], [8], avian *FoxP2* is required for normal song-learning in songbirds [12], [13], supporting the view that this is a molecule with broader relevance for vocal-learning in multiple species. Intriguingly, it has been independently proposed that specific changes in patterns of neural connectivity in the brains of vocal learners account for the differences in their speech/song behaviours relative to other closely-related species that lack such abilities [34]–[36]. Perhaps evolutionary differences in FoxP2 orthologues may contribute to altered patterns of connectivity in the different species, and thereby help to explain differential capacities for vocal learning. Since we did not assess the impact of evolutionary changes in the present study, this remains an open question for future investigation using comparative functional genomics.

To our knowledge, the current report represents the first large-scale *in vivo* characterisation of direct and indirect Foxp2 targets in the embryonic brain. It is of interest to consider how the present findings relate to published screens that used more limited ChIP surveys [19]–[21], or that employed expression profiling [22], [33]. The extent of direct overlap with previous datasets is complicated by three confounding factors. First, there are differences in scope of screening; the prior ChIP-chip investigations only queried a small subset of known promoters [20], [21]. Second, there are differences in species under investigation. Previous target screens largely focused on human and/or chimpanzee FOXP2, and the differences between the two [19]–[22], [33], while here we chose to comprehensively define the pathways regulated by murine Foxp2. Mouse models offer considerable advantages for functional genomics, and careful integration of murine data with those from other species will enhance our understanding of evolutionary roles of this gene. Finally, the majority of earlier studies screened neuron-like cells grown in culture [19]–[21], and no investigation of this transcription factor has reported integrated use of genome-wide ChIP and expression profiling to screen the same tissue.

Nevertheless, many important consistencies are observed between the different datasets, particularly in the biological themes and processes that they implicate. For example neurite outgrowth pathways and synaptic plasticity are over-represented in all FoxP2 ChIP-chip datasets across different species and neuronal cell-type, *in vitro* and *in vivo* [20], [21]. These processes are closely related during the development of neuronal networks. Genes controlling neurite outgrowth or axon guidance during early development have crucial roles in maturation and stabilisation of synaptic connectivity at later stages and eventually in activity-dependent synaptic plasticity in the mature brain throughout life (such as neurotrophins, semaphorins and ephrins) [37], [38]. Hence, the strong impact of Foxp2 on neurite outgrowth during one particular stage at E16 might even reflect major Foxp2 functions that are relevant throughout the development and maintenance of neuronal networks. A case in point is provided by our data demonstrating that *Nrn1* is a highly robust downstream target. The *Nrn1* gene encodes neuritin, which is already expressed at embryonic stages of development and was initially identified as a downstream effector of neuronal activity and neurotrophin-induced neurite outgrowth [28]. *Nrn1* not only showed one of the strongest enrichment signals in our *in vivo* ChIP experiments, but was independently detected as a target in our systematic expression profiling experiments of equivalent tissue and by *in situ* hybridisation – the corresponding human homologue was also one of the top direct targets reported in a small-scale ChIP screen of human foetal brain tissue [20].

A number of additional genes, which overlap with earlier studies, merit further comment. The *Cck* gene, which showed convergent evidence in our embryonic ChIP experiments, expression profiling screens and *in situ* hybridisation analyses, was reported as a direct target in both prior published human ChIP-chip studies [20], [21]. *Lmo4* (Lim domain only 4) was found to be indirectly downregulated by Foxp2 in our analyses of embryonic brain tissue (Table S4) and the human orthologue *LMO4* was similarly repressed by FOXP2 in previous expression profiling studies of human neuron-like cells by Konopka and colleagues [22]. Interestingly, in that earlier study using cellular models, this indirect target was repressed both by human and chimpanzee versions of FOXP2 [22]. *LMO4* encodes a transcription factor that plays important roles in cortical patterning, and is one of the few genes known to show asymmetric expression in the embryonic human brain [39]. *Efnb2* (Ephrin-b2), a well-validated direct target (Figure 1, Figure 4, Figure 5) was identified in the Konopka et al. study as one of a small number of genes that may be differentially regulated by human and chimpanzee FOXP2 orthologues [22]. This gene is of particular interest since it is implicated in neurite outgrowth and axon guidance (and also synaptic plasticity) in the basal ganglia and related brain structures [40]. In addition, *Nell2*, a validated ChIP and expression array target (Figure 3), has also been linked to neurite outgrowth [41], and has recently been shown to promote neuronal survival by *trans*-activation by estrogen [42].

Given the substantially enhanced scope of ChIP screening in the present study, we were able to identify many interesting novel targets that could not be isolated in the earlier work. For example, our high-confidence shortlist of direct targets includes *Pak3* – a downstream effector of the Rho family of GTPases which plays critical roles in pathways restraining neurite growth [43]; *Nptn* (neuroplastin) – encoding a synaptic glycoprotein involved both in development/maintenance of synaptic connections [44] and in long-term plasticity [45]; *Wasf1* – a gene that regulates activity-induced changes in dendritic spine morphogenesis [46] and is involved in actin remodelling during axon growth [47]; the neuronal semaphorins *Sema4f* [48] and *Sema6d* [49]; as well as *Ywhah* (also known as 14-3-3), which encodes an adapter protein implicated in presynaptic plasticity [50] (Figure 1, Figure 4, Figure 5; Table S3). Although the screening tissue was embryonic brain, many of the relevant genes have functions that go beyond this to also influence neural plasticity at later stages. Overall, this dataset will be important for directing follow-up studies of Foxp2-dependent pathways and assessing their involvement in traits such as acquisition of motor-skills [9], vocal learning [14], and spoken language [1]. While it is likely to be an indirect target of Foxp2 regulation, it is noteworthy that *Evf1/2* (*Shhrs*) showed such highly increased expression in *Foxp2-S321X* mice. It has been shown that the *Evf2* RNA molecule co-operates with the Dlx2 protein to activate the *Dlx5/6* enhancer element [27]. Thus it is interesting that both the *DLX1/2* and *DLX5/6* loci have been implicated in autism via independent studies, including a common polymorphism in the *DLX5/6* enhancer itself [51]–[53].

Of 340 genes showing differential expression (p<0.01) between mutant and wild-type ganglionic eminences, only 19 (∼5%) corresponded to putative direct targets of Foxp2 from the ChIP-chip screens. Thus, most of the expression differences observed in the transcriptional profiling experiments are unlikely to represent direct modulation due to Foxp2 binding, but could instead represent cascade effects further downstream (i.e. loss of Foxp2 directly alters expression of a relatively small subset of genes, which in turn indirectly affect many others). Discrepancies between the ChIP-chip and expression profiling datasets may also result from our experimental design: the former could potentially detect binding events of Foxp2-expressing neurons anywhere in the brain, while the latter was targeted specifically at the ganglionic eminences, a region showing particularly high Foxp2 levels. Foxp2 target genes that are not expressed in this structure could therefore be observed in the ChIP study, but would not be detected in the expression analysis. An example of such a target is *Sema3a*. The promoter of this gene was bound by Foxp2 in our ChIP study (Figure 1), but its expression only overlaps with Foxp2 expression in the cerebellum (Figure S6). Nevertheless, it is not unusual in studies of transcription factor function to observe substantial differences between promoter occupancy maps and transcriptional profiling data. It is well established that transcription factors can be poised ready at particular genomic sites, awaiting important co-factors, before modulating expression of the relevant targets [2], [54], [55].

The present investigation queried the vast majority of known promoters in the genome, but we acknowledge that the screening strategy is unable to uncover potential regulatory sequences that lie outside classical promoter regions. In earlier work, based on low-throughput shotgun sequencing of human FOXP2-ChIP fragments, we identified a FOXP2-bound element in the first intron of *CNTNAP2* (contactin-associated-protein-like-2) a gene implicated in language impairments and autism [19]. Although the mouse genome contains an orthologous region to the human FOXP2-bound regulatory element of *CNTNAP2*, this was not represented on the arrays used in this study, and hence it escaped detection. When we carried out ChIP-PCR experiments using the same mouse embryonic brain tissue as used for ChIP-chip we demonstrated clear Foxp2 occupancy of the orthologous region in mouse *Cntnap2*. Specific enrichment was observed in the wild-type brains; while no enrichment was found in equivalent tissue from the mutant null controls (see Figure S8 and Table S7). Studies are now underway using ‘ChIP-seq’ techniques (coupling ChIP to next-generation-sequencing) to allow a fully unbiased view of FOXP2/Foxp2 binding throughout the genome.

Among the validated direct targets of Foxp2 identified in our study there were a number of microRNA (miRNA) molecules, including *mir-124a* and *mir-137*. miRNAs are an extensive class of short (∼18–23 nucleotide) noncoding molecules which provide extra layers of dynamic control in networks of gene expression [56]. miRNAs are abundant in the brain and implicated in critical aspects of nervous system development and function, ranging from early neurogenesis and proliferation [57], through neural differentiation and dendrite morphogenesis [58], to adaptive mechanisms in mature neurons, including learning and memory [59]. They play pivotal roles in processes such as neurite outgrowth, axonal pathfinding and synaptic plasticity, mechanisms for which localised rapid control of protein synthesis is paramount [58], [59].

In conclusion, the use of *in vivo* genomic screening strategies in the developing embryonic brain has proved to be a powerful approach for understanding the biology of Foxp2, one of the most intriguing transcription factors of the CNS. This starting point led us to functional characterisation of new mechanisms of Foxp2 action, in particular the modulation of networks involved in neurite outgrowth, axonogenesis and other core aspects of neural development. Future studies will define how these regulatory networks differ between distinct species, what role miRNAs play in Foxp2-related pathways and phenotypes and will investigate whether it is possible to rescue the established neurobiological effects associated with loss of Foxp2 function, through manipulation of key targets. Ultimately, such work promises to fully uncover the functional pathways that connect Foxp2 with plasticity of the developing CNS.

## Materials and Methods

### In vivo chromatin immunoprecipitation

*In vivo* Foxp2-ChIP in embryonic mouse brain tissue was performed according to the protocol previously described by Vernes and colleagues [21]. Each of the three replicates included whole brain tissue (from the telencephalon to the brain stem at the level of the foramen magnum) at E16 (embryonic day 16), a developmental timepoint of high Foxp2 expression [16], pooled from 5–6 mice of matching genotype. Experiments were carried out either with wild-type embryos, or homozygous *Foxp2-S321X* mutants as negative controls. *S321X* mutants carry an early nonsense mutation that disrupts *Foxp2*; the resulting combination of nonsense-mediated RNA decay and protein instability leads to a complete lack of detectable Foxp2 protein in the brain [9]. The wild-type embryos and mutant controls used in these experiments were all matched littermates, backcrossed for at least ten generations into a C57BL/6J strain, maximizing the homogeneity of the genomic background. Although homozygous mutants display developmental delays and reduced cerebellar growth after birth, they show no gross anomalies in brain anatomy or development during embryogenesis [9]. All animal work was carried out conforming to the regulatory standards of the UK Home Office, under Project Licence 30/2016.

E16 mouse brains were extracted, snap frozen in liquid nitrogen and stored at −80°C until use. Each whole brain was weighed, then chopped finely with a razor on ice. Brains were pooled to achieve a total weight of between 0.3 and 0.5 g of tissue (between 5–6 brains per replicate) and resuspended in 5 ml PBS. A 1/10 volume (500 µl) of cross-linking buffer was added prior to 15 minutes incubation with agitation at room temperature. Formaldehyde was quenched via the addition of 125 mM glycine. Cross-linked tissue was washed in PBS before brief mechanical homogenisation. Pellets were then incubated in two *in vivo* ChIP lysis buffers at room temperature for ten minutes each: Buffer 1 (50 mM HEPES-KOH pH  = 7.5, 140 mM NaCl, 1 mM EDTA, 10% glycerol, 0.5% NP-40, 0.25% Triton X-100, protease inhibitors); Buffer 2 (200 mM NaCl, 1 mM EDTA, 0.5 mM EGTA, 10 mM Tris pH  = 8, protease inhibitors). After collection via centrifugation, nuclei were resuspended in 5 ml sonication buffer (10 mM Tris-HCl pH  = 8, 1 mM EDTA, 0.5 mM EGTA, protease inhibitors). Samples underwent 15 rounds of 20-second sonication pulses at 30% power, with 60 seconds on ice between each round (Branson Digital Sonifier - S450D). Agarose gel electrophoresis was used to confirm that fragment size was 300–1000 bp. Cells were centrifuged at 10,000 *g* and 4°C for 10 minutes to remove cell debris. 10 µg of polyclonal rabbit anti-Foxp2 antibody (C-terminal antibody, Geschwind Laboratory, UCLA) [20] pre-coupled to 100 µl Dynal M-280 rat anti-rabbit IgG magnetic protein-A beads was added and incubated at 4°C, rotating overnight. Beads were washed five times in RIPA buffer and once in TE buffer. Chromatin was eluted from beads in TE buffer with 1% SDS at 65°C for 10 minutes with agitation. The chromatin was then incubated at 65°C overnight to reverse cross-links. Purified chromatin was amplified via Ligation Mediated PCR (LMPCR) according to published protocols [60]. Size and purity of DNA was assessed via spectrophotometry and gel electrophoresis.

### Hybridisation of Foxp2-ChIP products to promoter microarrays

2 µg of amplified immunoprecipitated chromatin, or total input DNA was fluorescently labelled with Cy5 and Cy3 respectively using random primers provided in the BioPrime DNA labelling system (Invitrogen). The labelling reaction was allowed to proceed for 16 hours at 37°C, before purification by sodium acetate precipitation. Hybridisation to mouse promoter arrays (Agilent Technologies, #G4490A) was carried out by the UCLA microarray core facility, according to the manufacturer's instructions. Arrays consisted of 60-mer oligonucleotides spanning ∼8 kb (5.5 kb upstream and 2.5 kb downstream of TSS) at each of ∼17,000 mouse promoter regions. Probes were spaced on average, between 100–300 bp apart, with approximately 25 probes for each promoter region. Three littermate matched sets of pooled wild-type or mutant control chromatin samples were applied to microarrays, each using its respective input DNA sample as the internal reference on the array. Thus, the three wild-type and three mutant control datasets represent signals obtained from a total of 34 individual mouse embryos.

### Analysis of promoter microarray data

Array images were scanned using the Axon GenePix 4000B. Data were retrieved and initial quality control carried out using the Axon GenePix 4000B software package. All promoter coordinates and probes were mapped with reference to the NCBI m36 mouse assembly. Microarray data analysis was carried out using the mArray package for R [61]. LOESS normalisation and background correction was performed within each array. Data were normalised between arrays using quantile normalisation, and mean values were calculated from three biological replicates (wild-type or mutant control experiments) for each probe - called ‘probe scores’, such that a score of 1 corresponds to 2-fold enrichment in ChIP versus total input DNA. All negative probe scores were assigned a value of zero. A ‘window-adjusted score’ for each probe was then calculated as the median value of each probe score and its nearest neighbour on either side. Neighbouring probes were only considered if they fell within 500 bp upstream or 500 bp downstream of the central probe. This window size was based on the average size of the labelled DNA fragments, estimated to be approx 1000 bp. Thus, a true binding event would likely be indicated by positive scores of multiple neighbouring probes within a 1000 bp window. In cases where there were less than three probes located within this 1000 bp window showing a signal greater than background then the window-adjusted score was set to zero. This process helps to guard against artificial skewing of enrichment values at edges of promoter regions.

The use of mutant null controls enabled us to robustly assess the empirical significance of wild-type ChIP results. The data from the mutant control experiments were used to estimate a null distribution of window scores; that is, the non-specific signals produced by the Foxp2-ChIP protocol even when there is no Foxp2 protein available for pulldown. (Note that a subset of binding events in mutant null controls could potentially be due to crossreactivity of Foxp2 antibodies with closely related proteins, such as Foxp1 or Foxp4, that may bind to the same promoter. However, prior work with the antibody used here suggests that levels of crossreactivity are extremely low [20].) From this null distribution of window scores we calculated the threshold which excluded 99% of all datapoints in controls. This threshold could then be applied to the wild-type array data.

### Semi-quantitative PCR

Chromatin isolated during Foxp2-ChIP in mutant and wild-type mouse brains was amplified using a semi-quantitative PCR technique, as described previously [21], using primers directed towards the peak regions of enrichment (Table 3). The β-actin housekeeping gene promoter was used as a negative control.

**Table 3:** Semi-quantitative PCR primers.

GENE	FORWARD PRIMER (5′→3′)	REVERSE PRIMER (5′→3′)
Nrp2	CAGCAACCAGTGATGCTTGT	AGCAAGGAGAGTTGGAGCAA
Sema3a	GGGCACTGAGTAGCTTCCAC	GAATGCAAGAAAAGTTGTCCTC
Nrn1	TCCCCCAAACAAATTCTCAA	AATCCTTGCAGCATTTCAGG
β-actin	AGGGTACCACCGGAAAAGTC	CCCCAAAGGCTGAGAAGTTA

### Expression profiling

The ganglionic eminences, sites of particularly high embryonic Foxp2 expression [16], were dissected from E16 brains of six wild-type mice and six homozygous *Foxp2-S321X* mutant littermates. For each embryo, the left- and right-hemisphere ganglionic eminences were pooled in TRIzol reagent and RNA was extracted using the QIAGEN RNeasy kit, according to the manufacturer's instructions. RNA yield was measured using a NanoDrop ND-1000 spectrophotometer (NanoDrop Technologies, Wilmington, DE), and its quality was assessed using RNA6000 Nano Assays on an Agilent Bioanalyzer 2100 (Agilent Technologies, Santa Clara, CA).

Gene expression profiling was performed using whole-genome mouse BeadChip arrays from Illumina (San Diego, California, USA), which include 45,281 probes representing 31,492 mouse transcripts. In brief, 500 ng of total RNA was reverse transcribed to synthesize first- and second-strand complementary DNA (cDNA). Following purification on spin columns, *in vitro* transcription was used to synthesize biotin-labelled complementary RNA (cRNA). 1500 ng of biotin-labelled cRNA was hybridized to Mouse WG-6 V2 Expression BeadChip arrays (Illumina Inc., San Diego, CA) at 55°C for 18 h. The hybridized arrays were washed and labelled with streptavidin-Cy3 according to the manufacturer's protocols before being scanned with the Illumina Bead Array Scanner. Raw data were exported from the Illumina BeadStudio software (v3.4.0) for further processing and analysis using the R statistical software [62] and BioConductor packages [63]. Signal data and detection scores were extracted for each of the 12 samples. Signal data were background corrected by subtracting the average signal from the negative control probes on each array, prior to being transformed and normalised using the ‘VSN’ package [64]. Quality control analyses, including hierarchical clustering and principal component analysis (PCA), identified one outlier sample (from the wild-type group). This sample had very low signal compared to other samples while hybridisation and labelling metrics were normal, suggesting a sample problem rather than a technical issue. It was sufficiently outlying to remove from further analysis and the remaining 11 samples were re-normalised. The dataset was then filtered to remove probes that were not detected (detection score <0.95 in all samples), resulting in a final dataset of 24,479 probes.

Statistical analysis was performed using the ‘Linear Models for Microarray Analysis’ (Limma) package [65]. Differential expression between mutant and wild-type animals was assessed using a linear model that included effects for genotype and litter. Raw p-values were corrected for multiple testing using the false discovery rate (FDR) controlling procedure of Benjamini and Hochberg [66]. Fourteen probes were significant at a FDR of 5%. The larger set of 340 probes significant at p<0.01 was used for further biological investigation. We performed permutation tests on the genotype labels (11 choose 5), taking litter effects into account, and found that ≥340 genes were differentially expressed at p = 0.01 in only 9 out of the possible 108 permutations (∼8%). Gene annotation was added to the final probe list using the relevant annotation file (MouseWG-6_V2_0_R0_11278593_A.txt) from the Illumina website (http://www.illumina.com).

### Cell culture and reagents

Neuro2a (murine neuroblastoma) cells were cultured in ‘Growth media’: Modified Eagles Medium (MEM) (Sigma) supplemented with 10% Foetal Calf Serum (Sigma), 2 mM L-glutamine (Sigma) and 2 mM Penicillin/Streptomycin (Sigma). Cells were grown at 37°C in the presence of 5% CO_2_. Stable Neuro2a cell-lines overexpressing Foxp2 protein or non-expressing controls, were generated via transfection with pcDNA3.1/*Foxp2* (mouse isoform I - untagged) or the empty vector, using Genejuice (Novagen) according to the manufacturers' instructions. Cells were cultured in complete medium supplemented with 500 µg/ml G418 (Calbiochem) as a selective agent. Resistant single colonies were isolated 20 days after transfection, then cultured and expanded independently in the presence of G418 (500 µg/ml). Expression of recombinant Foxp2 was confirmed using qRT-PCR and Western blotting with two polyclonal antibodies recognizing different epitopes of the protein (goat N-terminal antibody, Santa Cruz Biotechnology [30]; rabbit C-terminal antibody, Geschwind Laboratory, UCLA [20]). Three Foxp2-transfected clones with a high and consistent level of expression and three empty vector clones were chosen for use in further experiments. Neuro2a cells were differentiated via the addition of Modified Eagles Medium supplemented with 2% Foetal Calf Serum (Sigma), 2 mM L-glutamine (Sigma), 2 mM Penicillin/Streptomycin (Sigma) and 20 µM all-trans retinoic acid (‘Differentiation media’).

### Quantitative RT-PCR

RNA was extracted from three independent clones of Neuro2a cells stably transfected either with murine *Foxp2* or the empty control vector following culture in growth media or differentiation media (for 24 or 48 hours). Total RNA was extracted from cells harvested in TRIzol reagent using the RNeasy kit (QIAGEN) according to manufacturers' instructions. Reverse transcription was performed as described previously [21]. Small molecular weight RNA was harvested using the Purelink miRNA isolation kit (Invitrogen) according to manufacturers' instructions. In order to assess miRNA expression levels, the small molecular weight RNA was polyadenylated prior to reverse transcription using the NCode miRNA First-strand cDNA synthesis kit (Invitrogen), as per the manufacturers' protocol.

PCR reactions utilised SYBR Green supermix (BioRad) as described [21]. Primers specific for candidate genes and the control housekeeping genes *GAPDH/Gapdh* (glyceraldehyde 3-phosphate dehydrogenase) and *U6* (small nuclear RNA) were designed using PrimerBank [67] (Table 4). Quantitative PCR reactions were performed on the iQ5 thermal cycler real-time PCR detection system (BioRad) according to manufacturers' instructions. Melting curve analysis was performed to assess the specificity of the amplification. Data analysis was performed using iCycler software (BioRad), and quantification was via the comparative CT method [68]. Fold changes are reported in response to Foxp2 expression compared to cells transfected with an empty vector, following normalisation to an internal control, the *GAPDH* housekeeping gene (for gene expression) or *U6* small nuclear RNA (for miRNA expression). Data are expressed as mean of values from three independent clones ± standard error of the mean (SEM). Statistical significance was assessed using Students t-tests (two-tailed).

**Table 4:** Quantitative RT-PCR gene-specific primers.

GENE	FORWARD PRIMER (5′→3′)	REVERSE PRIMER (5′→3′)
Acvr2a	ATAAACGGCGACATTGTTTTGC	TCAGTCCTGTCATAGCAGTTGA
Efnb2	ATTATTTGCCCCAAAGTGGACTC	GCAGCGGGGTATTCTCCTTC
Etv1	AGTTTGTACCAGACTATCAGGCT	CGGTGTATGAGTTGAGTTTGGA
Foxn2	ACTCCAGATAAGAGAGCTGAGAC	CTGCTGACTCACTGCCCAC
Nfat5	ATCGCCCAAGTCCCTGTACT	GCTTGTCTGACTCATTGATGCTA
Nptn	TCAGAACGAACCAAGAATTGTCA	AGAGCTGGAAGTGAGGTTACA
Nrn1	CCTGGACGACAAGACGAACAT	CCACATATCTTTCGCCCCTTC
Nrp2	AGGACACGAAGTGAGAAGCC	GGGGCGTAGACAATCCACTC
Sema6d	GGACTGCCACGGTAAGACAG	CAAATGCGGTCCAAAGGAGT
Wasf1	ATGCCGTTGGTGAAAAGAAACA	ACACTAACAGACAATCGGTCCA
Ywhah	ACGAAGATCGAAATCTCCTCTCT	CCGGTAGGCTTTAACTTTCTCCA
Foxp2	AAGCAGCTTGCCTTTGCTAAG	GGATTGAATGTATGTGTGGCTGA
Gapdh	TGACGTGCCGCCTGGAGAAAC	CCGGCATCGAAGGTGGAAGAGT
mmu-mir-124a-3	TAAGGCACGCGGTGAATGCC	Universal primer^a^
mmu-mir-137	TTATTGCTTAAGAATACGCGTAG	Universal primer^a^
mmu-let-7d	AGAGGTAGTAGGTTGCATAGT	Universal primer^a^
mmu-mir-9-2	TCTTTGGTTATCTAGCTGTATGA	Universal primer^a^
mmu-mir-216	TAATCTCAGCTGGCAACTGTGA	Universal primer^a^
U6	CTGCGCAAGGATGACACG	Universal primer^a^

**a** miRNA and U6 reactions used the Universal qPCR reverse primer supplied with the NCode miRNA First-strand cDNA synthesis kit (Invitrogen).

### Primary cell culture

Ganglionic eminences from both hemispheres were dissected from wild-type and homozygous *Foxp2-R552H* E16 littermates [9]. *R552H* mice carry a missense mutation affecting a conserved arginine residue located in the Foxp2 DNA binding domain, matching an aetiological mutation found in a well-characterised multigenerational family with speech and language disorder (the KE family) [4]. *R552H* homozygous mice demonstrate comparable phenotypes to homozygous Foxp2 knockouts [9]. This suggests that the introduction of this mutation yields a stable, but non-functional protein product, a conclusion that is supported by *in vitro* functional studies [30]. Dissections were performed in dissection buffer (15 mM HEPES, 0.01% NaHCO_3_, 25 mM glucose in HBSS-CMF) and dissected regions were immediately chopped on ice and pelleted at 800 RPM and 4°C for 5 minutes. The pellet was incubated in papain solution (20 units/ml papain, 1 mM L-cysteine, 0.5 mM EDTA, 100 units/ml DNaseI, in dissection buffer) on ice for 5 minutes then at 37°C for 10 minutes, agitating regularly. The enzymatic reaction was halted by addition of Ovo-BSA solution (10 mg/ml ovomucoid, 10 mg/ml BSA in dissection buffer). Cells were pelleted at 1000 RPM for 5 minutes at 4°C and the pellet was washed then re-suspended in complete medium (neurobasal media (Sigma) supplemented by 2 mM Glutamax (Sigma). 2 mM Penicillin/Streptomycin (Sigma) and 1X B27 supplement). Suspension was triturated using plastic and glass pipettes to dissociate any remaining cell clumps before passing the cell suspension through a 70 µm cell strainer. Single cell suspensions were seeded onto laminin and poly-D-lysine coated coverslips (BD Biosciences) at a density of 6.3×10^4^ cells per well into 24 well plates and grown at 37°C in the presence of 5% CO_2_ in complete medium.

After 4 days in culture, cells were fixed using 4% Paraformaldehyde solution for 15 minutes at room temperature and permeablised in wash solution (0.1% Triton X-100 in TBS). Antibodies were diluted in Blocking Solution (1% Fish Gelatine, 0.1% Triton X-100, 5% BSA in PBS). Cells were co-stained at 4°C overnight, using two primary antibodies; an anti-MAP2 rabbit polyclonal antibody (Chemicon) and an anti-Foxp2 mouse monoclonal antibody recognising an epitope near the C-terminal end of the protein (Gift from Prof. A. Banham). Detection was then facilitated via four rounds of antibody incubation, which allowed magnification of the Foxp2 signal. Cells were incubated with anti-rabbit TRITC (Alexa Fluor 568, Molecular Probes) plus anti-mouse biotinylated (BA9200, Vector Labs) secondary antibodies, followed by incubation with anti-rabbit TRITC plus anti-biotin FITC (Alexa Fluor 488, Molecular Probes) antibodies, each for 1 hour, shaking under limited light exposure. This secondary/tertiary antibody incubation was then repeated under the same conditions. Nuclei were visualised using mounting media containing a DAPI counterstain (VectaShield). Cells were viewed on a Nikon Eclipse TE2000U fluorescence inverted microscope. Images were captured using a Hamamatsu black and white C4742-95 Orca hi-sensitivity CCD camera with IPLab imaging software (Scanalytics Inc), and analysed using the neurite outgrowth function of Metamorph Version 7.6 (Molecular Devices). Statistical analyses were carried out using ANCOVA (analysis of covariance) for genotype and embryo, followed by post-hoc Sidak correction. Data are expressed as the mean ± standard error of the mean (SEM).

### *In situ* hybridisation

*In situ* hybridization was carried out on 10 µM frozen sections of E16.5 embryo heads as previously described [69]. An approximately 500 bp fragment of each target transcript was subcloned into pCR4-TOPO (Invitrogen) for dioxygenin-labelled riboprobe synthesis. Primer sequences for the riboprobes are available on request. Equivalent parasagittal sections were hybridized in parallel from three wild-type and three homozygous *Foxp2-S321X* mutant embryos and all slides were developed for 16 hours, or 6 hours in the case of *Shhrs.* In all cases a sense-strand negative control riboprobe gave no specific signal (data not shown).

## Supporting Information

Figure S1 Western blot analysis of endogenous Foxp2 protein.
(TIF)

Figure S2 Chromosome distribution of enriched probes.
(TIF)

Figure S3 Window scores for all probes that exceeded the strict threshold (enrichment score of >1.5) in wild-type Foxp2-chip.
(TIF)

Figure S4 EMSA analysis of a novel Foxp2 DNA-binding motif.
(TIF)

Figure S5 Differential expression of Foxp2 target genes in E16 developing mouse ganglionic eminences.
(TIF)

Figure S6 Co-expression of Foxp2 and putative target genes in the E16 mouse brain.
(TIF)

Figure S7 Expression of Foxp2 in medium spiny neurons (MSNs).
(TIF)

Figure S8 *In vivo* binding of mouse Foxp2 to intron 1 of *Cntnap2* in embryonic brain tissue.
(TIF)

Table S1 Foxp2-ChIP (window score ≥0.97) complete dataset.
(XLS)

Table S2 Foxp2-ChIP putative target promoters (curated long list n = 1164, window score ≥0.97).
(XLS)

Table S3 Foxp2-ChIP putative target promoters (curated short list n = 264, at least 1 window score ≥1.50).
(XLS)

Table S4 Foxp2-dependent gene expression changes in the E16 mouse brain (p<0.01).
(XLS)

Table S5 Overlapping target genes from Foxp2-ChIP and expression analysis studies.
(XLS)

Table S6 Significant over-represented KEGG pathways identified in the Foxp2-ChIP and gene expression datasets.
(XLS)

Table S7 Cntnap2 semi-quantitative PCR primers.
(XLS)

Text S1 Supporting Materials and Methods.
(DOC)
