# Supplementary figures and images for: Foxp2 Regulates Gene Networks Implicated in Neurite Outgrowth in the Developing Brain
Source: PLoS Genet. 2011 Jul 7;7(7):e1002145. doi: 10.1371/journal.pgen.1002145 (PMC3131290; doi:10.1371/journal.pgen.1002145)

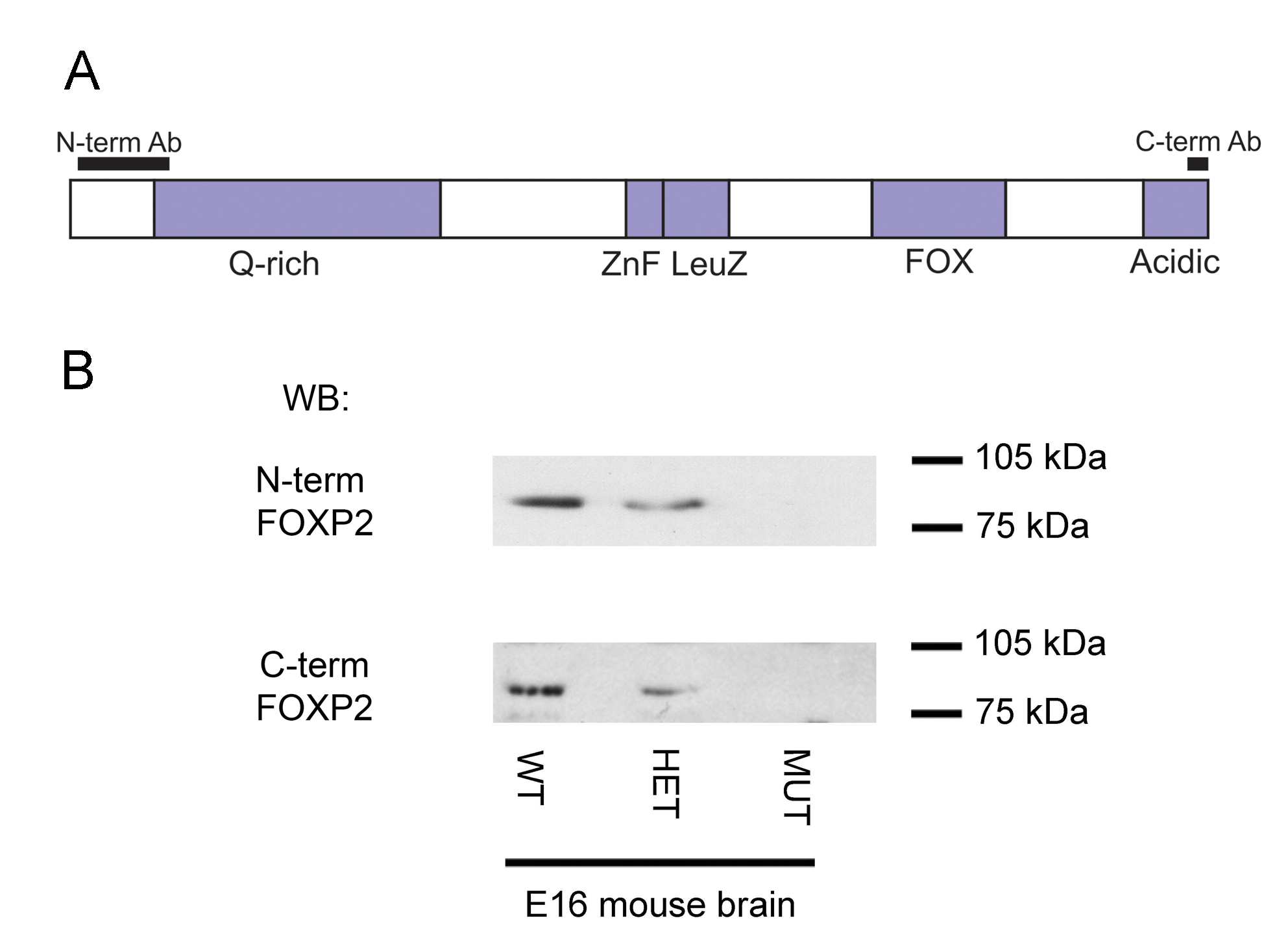

Supplement: Figure S1 — Western blot analysis of endogenous Foxp2 protein. (TIF) [file pgen.1002145.s001.tif]

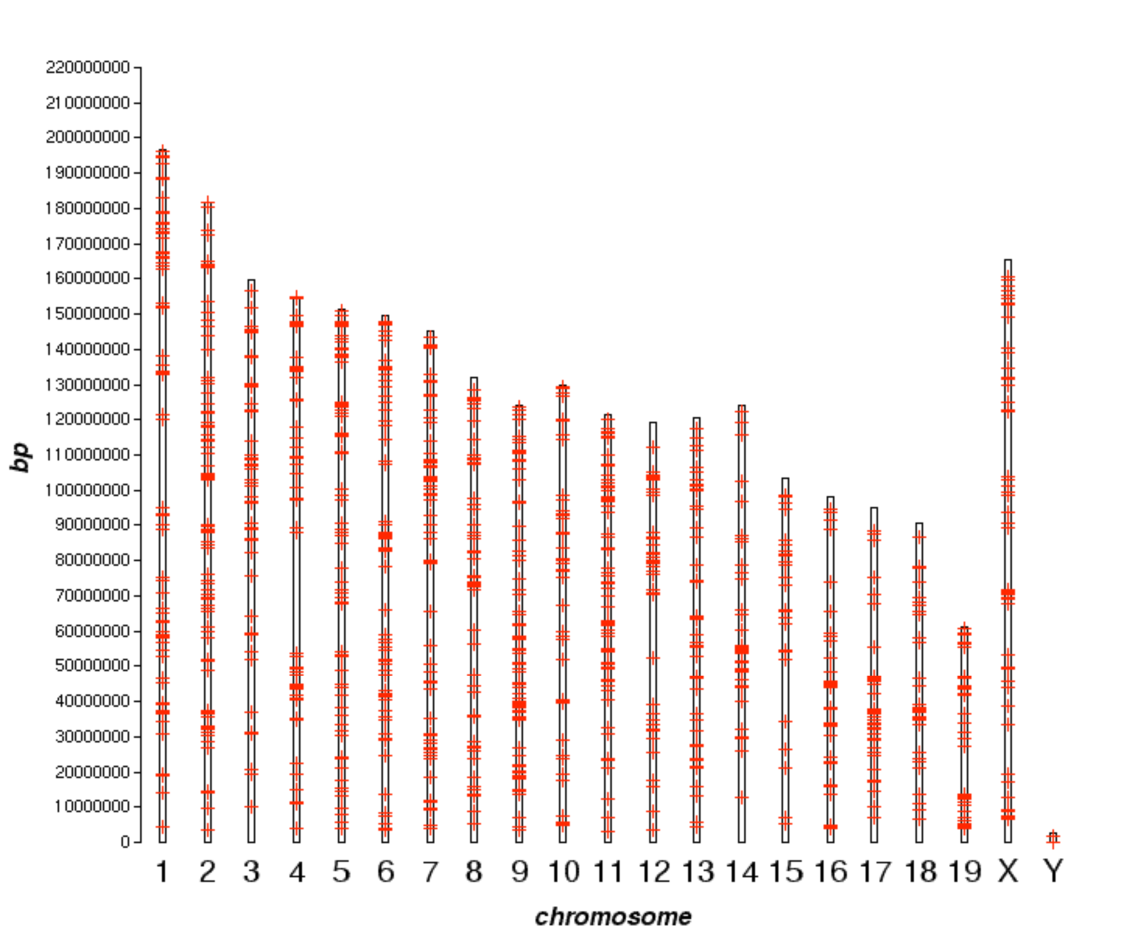

Supplement: Figure S2 — Chromosome distribution of enriched probes. (TIF) [file pgen.1002145.s002.tif]

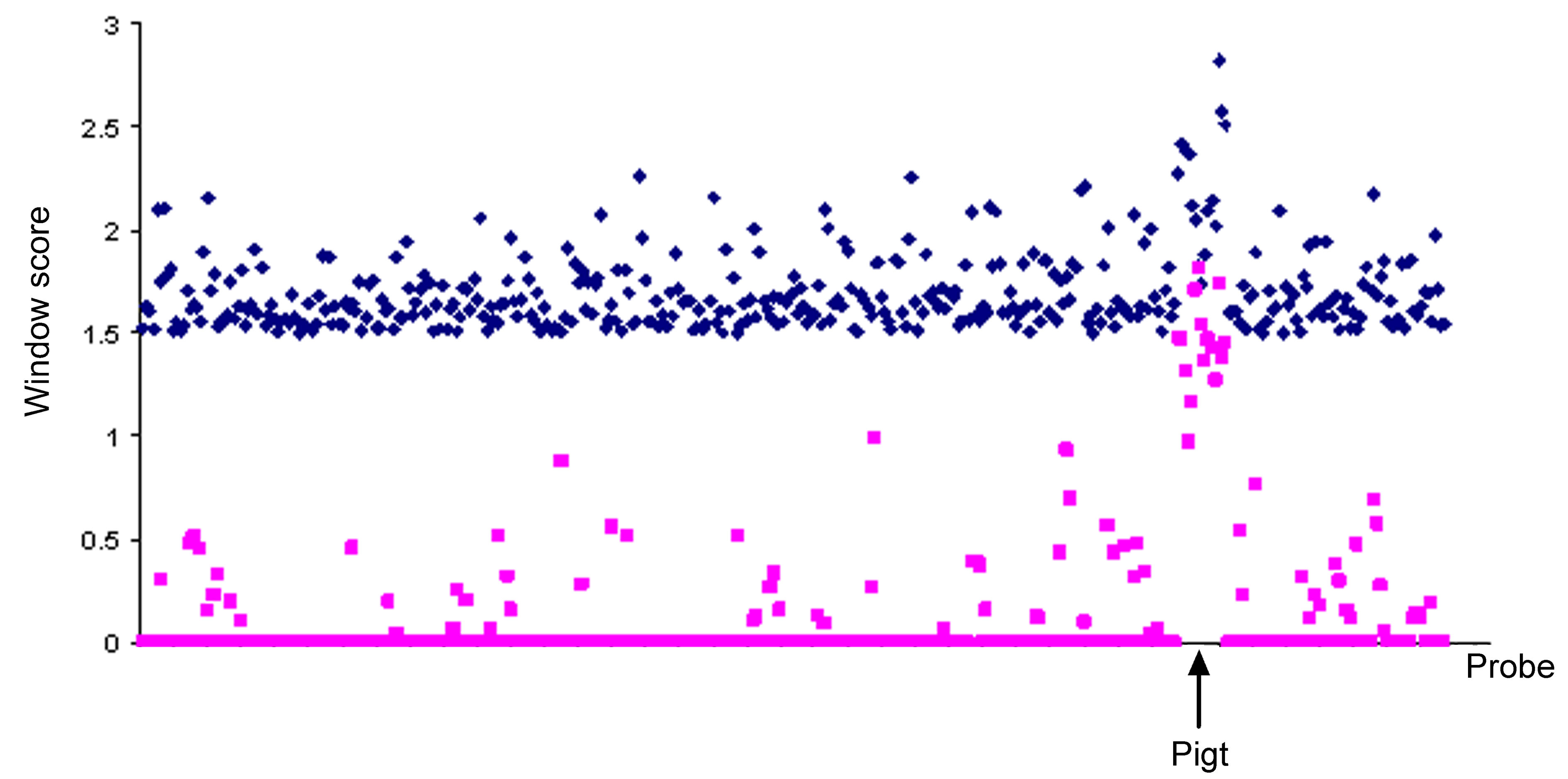

Supplement: Figure S3 — Window scores for all probes that exceeded the strict threshold (enrichment score of >1.5) in wild-type Foxp2-chip. (TIF) [file pgen.1002145.s003.tif]

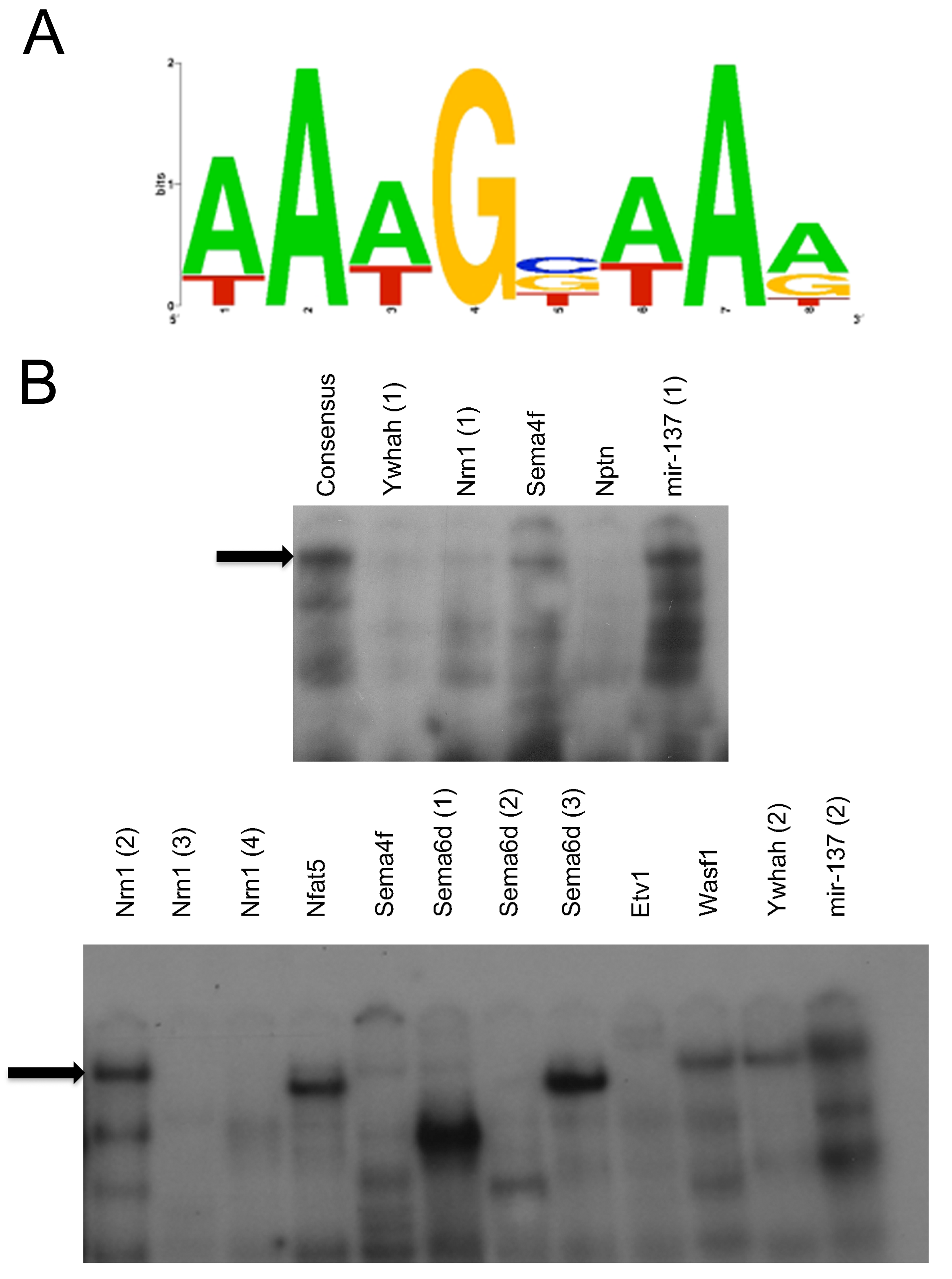

Supplement: Figure S4 — EMSA analysis of a novel Foxp2 DNA-binding motif. (TIF) [file pgen.1002145.s004.tif]

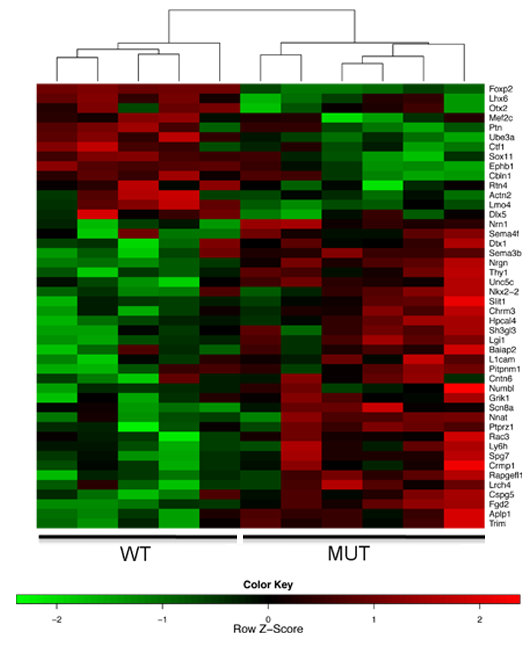

Supplement: Figure S5 — Differential expression of Foxp2 target genes in E16 developing mouse ganglionic eminences. (TIF) [file pgen.1002145.s005.tif]

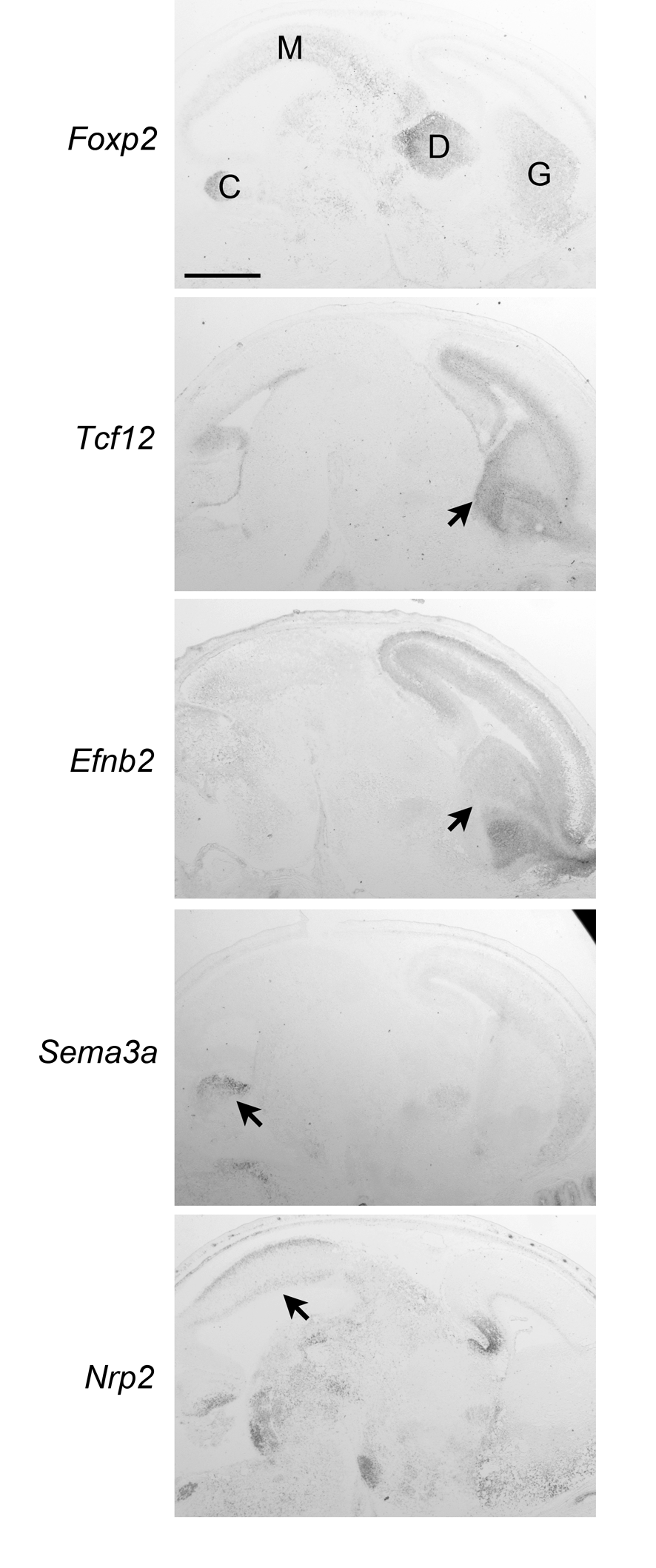

Supplement: Figure S6 — Co-expression of Foxp2 and putative target genes in the E16 mouse brain. (TIF) [file pgen.1002145.s006.tif]

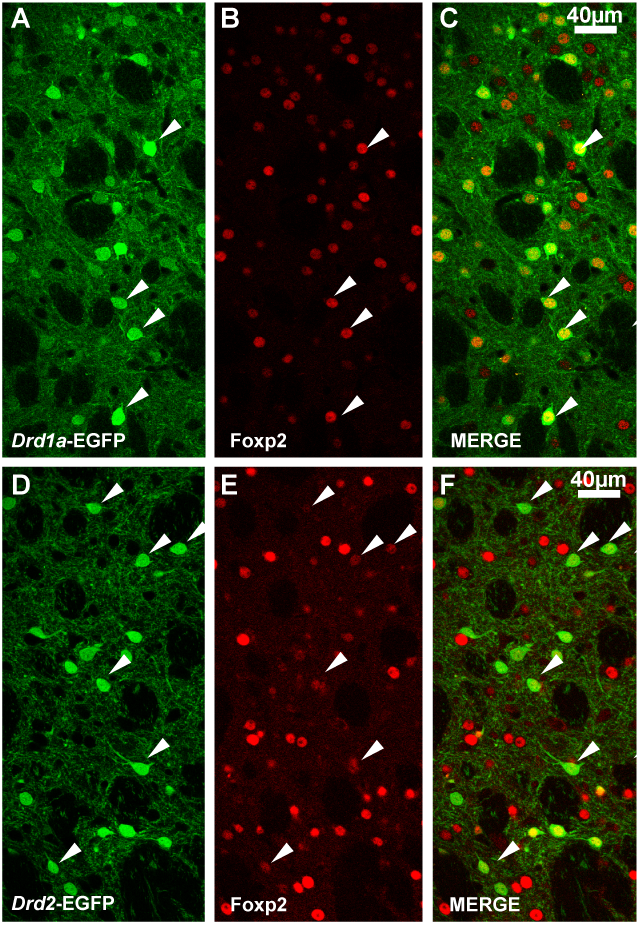

Supplement: Figure S7 — Expression of Foxp2 in medium spiny neurons (MSNs). (TIF) [file pgen.1002145.s007.tif]

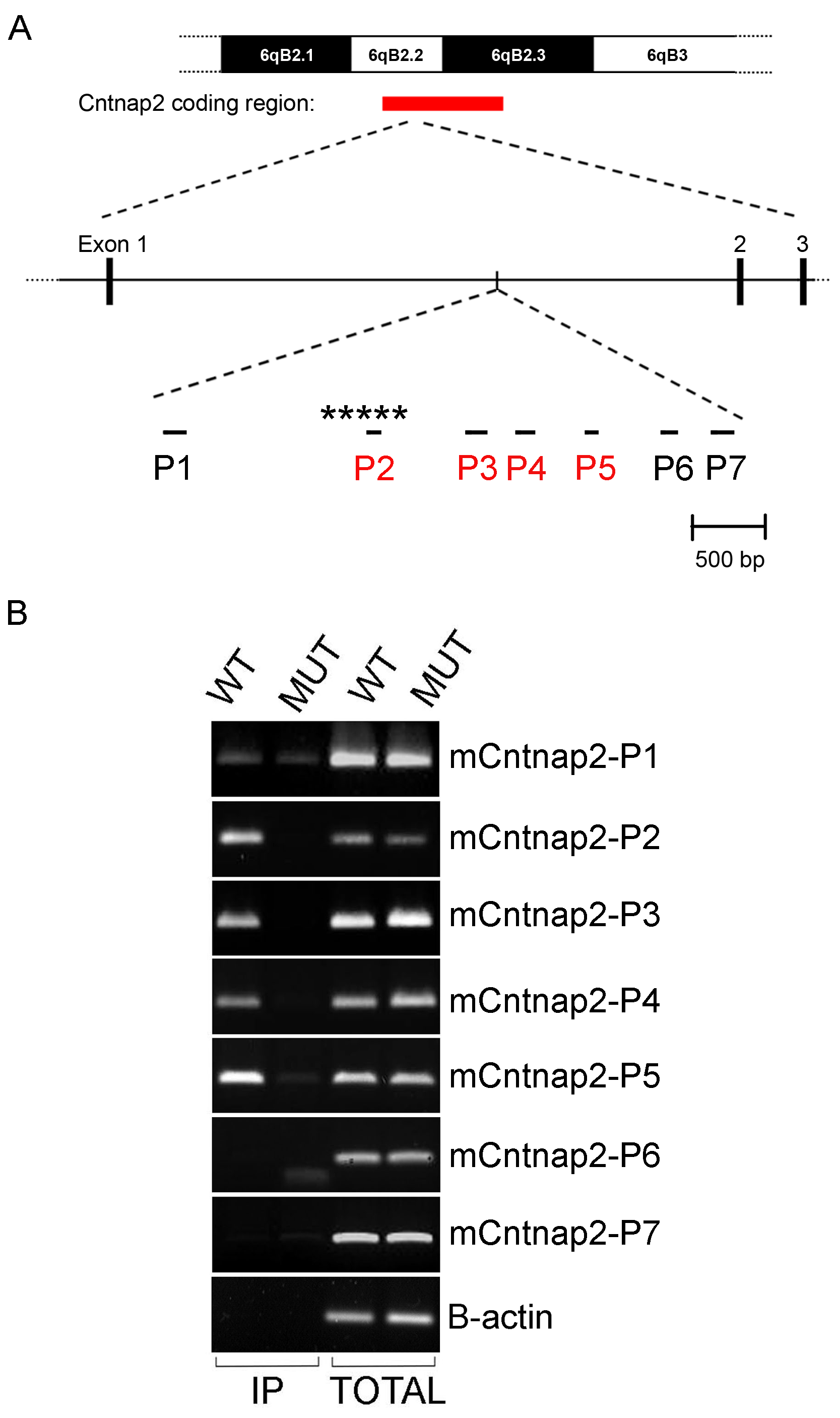

Supplement: Figure S8 — In vivo binding of mouse Foxp2 to intron 1 of Cntnap2 in embryonic brain tissue. (TIF) [file pgen.1002145.s008.tif]
