# Supplementary material for: Foxp2 Regulates Gene Networks Implicated in Neurite Outgrowth in the Developing Brain
Source: PLoS Genet. 2011 Jul 7;7(7):e1002145. doi: 10.1371/journal.pgen.1002145 (PMC3131290; doi:10.1371/journal.pgen.1002145)
Supplement: Text S1 — Supporting Materials and Methods. (DOC) [file pgen.1002145.s016.doc]

**TEXT S1**

**SUPPORTING MATERIALS AND METHODS**

**ChIP-chip microarray analysis – shortlist generation**

By taking only those promoters where at least one window score exceeded 1.5, a high-confidence shortlist was prepared, comprising 259 promoters (corresponding to 266 genes, as some promoters are bidirectional). For each probe with a score of ≥1.5 in the wildtype dataset we compared the level of enrichment observed for the corresponding probe in the mutant null control dataset (Figure S3). Only sixteen of the probes in this list had enrichment scores exceeding 0.974 (the 99% cut-off) in the null control dataset, fifteen in the *Pigt* promoter, and one in the *Zfp496* promoter. These findings highlight the very different pattern of enrichment observed in the wildtype experiments as compared to their null control counterparts. Since the data suggested that *Pigt*, and possibly *Zfp496*, represent false positives, we excluded these two genes from further consideration, and produced a short curated dataset of 264 high-confidence targets (Table S3).

**Mouse ChIP-chip motif analysis**

Sequences corresponding to the peak regions from the high confidence target promoters identified via Foxp2-ChIP (Table S3) were retrieved using the Ensembl Perl API. This analysis returned sequences for 247 of the 259 promoter regions, spanning each enriched probe (window score >0.974) plus the neighbouring probe on either side (usually ~1000 bp). The sequences used to carry out the analysis corresponded to the NCBI m36 mouse assembly. Bioprospector [1] was used to perform an unbiased search for any over-represented motifs of 8 bases in length, compared to the whole genome background probability of observing these nucleotides, a maximum of 40 times. One hundred motifs were returned, of which 26 were significantly enriched (p<0.05). The 26 significant motifs actually represented variations on 8 core sequences and as such were collapsed into these 8 motifs (motif A-H), as shown in Table 1 of the main text.

**DNA binding assays**

The ability of Foxp2 to interact with the new putative binding motif (motif D: AAAG[C/G]AAA) was assessed using Electrophoretic Mobility Shift Assays (EMSAs) according to published protocols [2]. Probes were designed as oligomers, 24 nucleotides in length. The consensus probe used was as follows (with the classical FOXP binding site underlined): 5’-agctttatttatgttgttttgtat-3’. The motif D probes were designed as follows (with the new putative motif, Motif D underlined):

Ywhah (1): 5’- AGCTTTTTAAAAAGCAAAAACAAA -3’

Ywhah (2): 5’- AGCTTTAAAATAAGCAAAATAAAT -3’

Nrn1 (1): 5’- agctAAAATTAAAGGAAAAGGCAA -3’

Nrn1 (2): 5’- AGCTATCCAAAATGCAAATGTTTT -3’

Nrn1 (3): 5’- AGCTTGGACAAAAGGAAAAAAATA -3’

Nrn1 (4) 5’- AGCTTTTTGCAAAGCAAGGAGCAT -3’

Sema4f: 5’- agctTTTTAAAAAGCTAAAAACTG -3’

Nptn: 5’- AGCTCTGAGCAAAGCAATATCCTT -3’

mmu-mir-137 (1): 5’- AGCTGGCAATTAAGCTAAAGCAGA -3’

mmu-mir-137 (2): 5’- AGCTTGAAATTATGTAAGTGCAAA -3’

Nfat5: 5’- AGCTAAACCCAATGTAAAGGTGGA -3’

Sema6d (1): 5’- AGCTACATCCTAAGCAAGATAAAT -3’

Sema6d (2): 5’- AGCTTGAGCCAATGTTAGTGCCTA -3’

Sema6d(3): 5’- AGCTGAGCTGAATGGTATGAAGTG -3’

Etv1: 5’- AGCTGCTGGAAAAGGTAACTTGGG -3’

Wasf1: 5’- AGCTGAAAAAAATGTAAATAGAAT -3’

**Expression profiling – heatmap generation.**

After initial GO analyses highlighted neurite-related phenotypes as a major biological theme, a heatmap was generated to focus on the relevant pathways, using the TIGR Multiexperiment Viewer (http://www.tm4.org/mev.html), based on the standardised residuals after fitting an effect for litter. This heatmap contained any significant genes from expression profiling (p<0.05) that were implicated in neurite outgrowth and development pathways via annotation with one or more of the following GO terms (Nervous System Development, Generation of Neurons, Neurogenesis, Neuron Development, Neuron Differentiation, Cellular Morphogenesis during Differentiation, Cell Projection Biogenesis, Neurite Development, Axonogenesis and Axon Guidance).

**Western blotting**

Proteins were extracted from E16 mouse brains and analysed for Foxp2 expression using western blotting carried out as previously described [2]. Two antibodies directed towards Foxp2 were used; a goat polyclonal, which recognised an epitope at the N-terminus (Santa Cruz Biotechnology) of isoform I and a rabbit polyclonal, which recognises a C-terminal epitope [3] (see Figure S1A). Anti-goat (DAKO) and anti-rabbit (BioRad) HRP-conjugated secondary antibodies were used to detect proteins.

**SUPPORTING REFERENCES**

1. Liu X, Brutlag DL, Liu JS (2001) BioProspector: discovering conserved DNA motifs in upstream regulatory regions of co-expressed genes. Pac Symp Biocomput: 127-138.

2. Vernes SC, Spiteri E, Nicod J, Groszer M, Taylor JM, et al. (2007) High-throughput analysis of promoter occupancy reveals direct neural targets of FOXP2, a gene mutated in speech and language disorders. Am J Hum Genet 81: 1232-1250.

3. Spiteri E, Konopka G, Coppola G, Bomar J, Oldham M, et al. (2007) Identification of the transcriptional targets of FOXP2, a gene linked to speech and language, in developing human brain. Am J Hum Genet 81: 1144-1157.

4. Groszer M, Keays DA, Deacon RM, de Bono JP, Prasad-Mulcare S, et al. (2008) Impaired synaptic plasticity and motor learning in mice with a point mutation implicated in human speech deficits. Curr Biol 18: 354-362.

5. Zhang B, Kirov S, Snoddy J (2005) WebGestalt: an integrated system for exploring gene sets in various biological contexts. Nucleic Acids Res 33: W741-748.

6. Vernes SC, Newbury DF, Abrahams BS, Winchester L, Nicod J, et al. (2008) A functional genetic link between distinct developmental language disorders. N Engl J Med 359: 2337-2345.

**SUPPORTING FIGURE LEGENDS**

**Figure S1 - Western blot analysis of endogenous Foxp2 protein.** (A) Schematic figure of FOXP2 isoform I demonstrating the position of epitopes recognised by the N-terminal (goat N-terminal antibody, Santa Cruz Biotechnology) [2] and C-terminal (rabbit C-terminal antibody, Geschwind Lab) [3] antibodies. (B) Proteins were resolved on SDS PAGE and detected using these N- and C-terminal antibodies. Samples were cell lysates of E16 mouse brain tissue isolated from homozygous wildtype (WT), heterozygous (HET) or homozygous mutant (MUT) *Foxp2-S321X* littermates. *Foxp2-S321X* heterozygotes showed reduced Foxp2 protein levels while no protein was detected in homozygous mutant samples, consistent with previous reports [4]. The lack of Foxp2 signal in the homozygous sample using both N- and C-terminal antibodies suggests that there is little or no truncated product present in the E16 brain (likely due to a combination of nonsense-mediated RNA decay and protein instability). The C-terminal antibody required much shorter exposure times to detect Foxp2 than the N-terminal antibody and thus was predicted to have a higher affinity. For this reason we chose to use the C-terminal antibody in the Foxp2-ChIP experiments.

**Figure S2 - Chromosome distribution of enriched probes.** The positions of all genes from wildtype Foxp2-ChIP that demonstrated promoter enrichment scores ≥0.974 (99% empirical threshold established from null controls) were plotted onto a chromosome map, using the WebGestalt program [5]. Gene locations are indicated by red bars. Distance in base pairs is given on the Y axis (bp). No overt positional bias was observed in the target gene list.

**Figure S3 - Window scores for all probes that exceeded the strict threshold (enrichment score of >1.5) in wildtype Foxp2-chip.** Wildtype data points are given in blue, with window scores for the corresponding probes in the null mutant control experiments shown in pink. The vast majority of strictly selected probes displayed dramatically reduced enrichment scores in the null control dataset, indicating that they represent robust Foxp2-specific binding events. Only sixteen of the enriched probes displayed window scores exceeding 0.974 in the null controls, fifteen of which were found in the *Pigt* promoter (arrow). The sixteenth probe was located in the promoter of the *Zfp496* gene. These two genes were excluded from the final high-confidence shortlist of ChIP-chip targets, since they may have been detected due to non-specific effects, independent of Foxp2 pulldown (see Table S2). None of the probes on the genome-wide arrays displayed an enrichment score of >1.5 in the null control ChIP-chip experiments. Probes are ordered with respect to their location in the genome (not to scale).

**Figure S4 – EMSA analysis of a novel Foxp2 DNA-binding motif.** (A) A novel putative FOXP2 binding site, here referred to as ‘Motif D’ (AAAG[C/G]AAA) was identified in 182 of the 247 retrieved Foxp2-ChIP enriched shortlist of promoters. (B) Nine target promoters containing Motif D were used to design probes for EMSA experiments to determine if the Foxp2 protein was able to bind this novel sequence. Radio-labelled probes were combined in binding reactions with nuclear extracts taken from Neuro2a cells stably transfected with Foxp2. Arrows indicate location of protein-DNA complexes, indicating successful binding of probe. 'Consensus' represents a probe containing a known Foxp2 binding site (TATTTAT).

**Figure S5 - Differential expression of Foxp2 target genes in E16 developing mouse ganglionic eminences.** Heatmap of genes implicated in neurite outgrowth pathways showing evidence of differential expression in Foxp2 knockout mice. The set of genes with p<0.05 in Foxp2 expression analysis that were also annotated with one or more relevant GO terms related to neuron development and neurite outgrowth are shown (see Supporting Materials and Methods for full list). Red indicates relatively high expression and green indicates relatively low expression in the corresponding samples. The standardised residuals, after fitting an effect for litter, were used to generate the heatmap.

**Figure S6 – Co-expression of Foxp2 and putative target genes in the E16 mouse brain.** *In situ* hybridisation of wildtype E16 brains for selected Foxp2 targets identified by Foxp2-ChIP. All genes show co-expression in at least one region of high *Foxp2* expression, including the ganglionic eminence (G), midbrain (M) and developing cerebellum (C). Scale bar is 1 mm.

**Figure S7 – Expression of Foxp2 in medium spiny neurons (MSNs).** Differential expression of Foxp2 in striatonigral and striatopallidal medium spiny neurons using GENESAT Drd1a-EGFP and Drd2-EGFP mouse lines, respectively. Confocal sections of the dorsal striatum, showing immunofluorescence (red; B, E) for Foxp2 alone and EGFP fluorescence (green; A, D) or the combination of Foxp2 and EGFP (C, F). Almost 95% of medium spiny neurons expressed Foxp2 (data not shown). Striatonigral MSNs labelled with EGFP exhibit high levels of Foxp2 (A, B, C). ­Conversely, striatopallidal MSNs labelled with EGFP display low levels of Foxp2 (A, B, C). Scale bars: 40 µm.

**Figure S8 -**- ***In vivo* binding of mouse Foxp2 to intron 1 of *Cntnap2* in embryonic brain tissue.** (A) The region orthologous to the clone previously isolated via human FOXP2 Shotgun-ChIP (detailed in reference [6]), was identified in the mouse genome, mapping within intron 1 of *Cntnap2* (located on 6qB2.2). Primers to test ChIP enrichment (mCntnap2-P1 - mCntnap2-P7) were designed to amplify small fragments spanning this region (see Table S7). Asterisks indicate a region of high conservation with human sequence (i.e. homologous to human clone). (B) Foxp2-ChIP was performed in E16 brain samples from wildtype mice (WT) or homozygous *Foxp2-S321X* mutant littermates (MUT) as described in the main text Materials and Methods. DNA was PCR-amplified using primers mCntnap2-P1-P7. Regions represented by mCntnap2-P2 to mCntnap2-P5 were specifically enriched in Foxp2-ChIP samples isolated from wildtype brains compared to those from mutant brains (highlighted in red font in part A). Flanking regions (mCntnap2-P1, P6, P7) were not specifically enriched. The β-actin promoter acts as a negative enrichment control.
